# Dysregulation of CircZNF79(5) Modulates YBX1 Stability and Selective Autophagy to Drive Hepatocellular Carcinoma Progression

**DOI:** 10.1002/advs.202510310

**Published:** 2025-11-12

**Authors:** Xueqiang Guo, Lingling Xi, Yukun Liu, Wenbao Lv, Tianzi Li, Andong Ju, Zhenlin Fan, Yaping Shen, Zhuang Qian, Weiyun Wang, Zhuo Liang, Wenjuan Song, Kaiwen Chang, Shuangping Ma, Junhe Zhang, Tao Han, Kun You, Cunshuan Xu, Lei Wang, Weisheng Guo, Wenjie Ren

**Affiliations:** ^1^ The First Affiliated Hospital of Xinxiang Medical University Institutes of Health Central Plain The Third Affiliated Hospital of Xinxiang Medical University Xinxiang Key Laboratory for Tumor Drug Screening and Targeted Therapy Xinxiang Key Laboratory of Molecular Oncology Xinxiang Medical University Xinxiang 453003 P. R. China; ^2^ State Key Laboratory of Antiviral Drugs Pingyuan Laboratory Henan Normal University Xinxiang 453007 P. R. China; ^3^ Abdominal Surgical Oncology Xinxiang Central Hospital Institute of the Fourth Affiliated Hospital of Xinxiang Medical University Xinxiang 453003 P. R. China; ^4^ Clinical Medical Center of Tissue Engineering and Regeneration Xinxiang Medical University Xinxiang 453003 P. R. China; ^5^ The First Affiliated Hospital of Xinxiang Medical University Xinxiang Medical University Xinxiang 453003 P. R. China; ^6^ College of Life Science Henan Normal University Xinxiang 453003 P. R. China; ^7^ Department of Minimally Invasive Interventional Radiology The Second Affiliated Hospital School of Biomedical Engineering Guangzhou Medical University Guangzhou 510260 P. R. China

**Keywords:** BRCC36, circZNF79(5), HCC, p62, selective autophagy, YBX1

## Abstract

Hepatocellular carcinoma (HCC) is a prevalent and aggressive liver malignancy with limited therapeutic options. Circular RNAs (circRNAs) have emerged as critical regulators in various cancers, including HCC, but their roles in HCC progression remain largely unexplored. Here, the role of circZNF79(5) in HCC progression and its underlying mechanisms is investigated. CircZNF79(5) expression in HCC tissues, cell lines and the serum exosomes is analyzed using qRT‐PCR and FISH, and evaluated its effects on cell proliferation, migration, invasion and apoptosis using CCK8, colony formation, EdU, Transwell and Flow cytometry. CircZNF79(5)’s is verified to be upregulated in HCC, and found that it can promote HCC cells proliferation, migration and invasion, while inhibiting the apoptosis. Mechanistically, circZNF79(5) is found to stabilizes the oncogenic protein YBX1 by recruiting BRCC36, a K63 chain deubiquitinating enzyme, thereby preventing YBX1 from p62‐mediated selective autophagic degradation via the AMPK/mTOR signaling pathway. In vivo studies using subcutaneous and orthotopic tumor models confirmed that circZNF79(5) knockdown reduced tumor growth and YBX1 expression. The findings reveal a novel mechanism by which circZNF79(5) promotes HCC progression through YBX1 stabilization and selective autophagy regulation, highlighting the circZNF79(5)‐YBX1‐BRCC36 axis as a potential therapeutic target for HCC.

## Graphical abstract

In HCC, circZNF79(5) binds to YBX1 and functions as an oncogene, recruits BRCC36 to remove K63‐linked ubiquitin chains to stabilize YBX1 protein, and promotes HCC progression via the HIF‐1 signaling pathway. Conversely, circZNF79(5) silencing activates the AMPK/mTOR pathway, inducing p62‐mediated selective autophagic degradation of YBX1. Furthermore, this study highlights the circZNF79(5)‐YBX1‐BRCC36 axis as a potential therapeutic target in HCC.

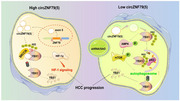

## 1 Introduction

Hepatocellular carcinoma (HCC) is a prevalent primary malignancy with a high mortality rate, posing a significant threat to human health and safety.^[^ ^1^ ^]^ Current clinical treatments for HCC primarily involve surgical resection and/or chemotherapy. However, these approaches often result in suboptimal long‐term outcomes. Moreover, the multifocal development and frequent distant metastasis observed in most patients further complicate the complete eradication of HCC, rendering the current diagnostic and therapeutic landscape highly challenging.^[^ ^2^ ^]^ Therefore, it is imperative to explore novel mechanisms underlying HCC progression to identify potential diagnostic and therapeutic targets.

Circular RNA (circRNA), a unique class of RNA molecules that form covalently closed loops through reverse splicing of linear RNA, has garnered significant attention due to its remarkable stability, sequence conservation, and tissue‐specific expression.^[^ ^3^, ^4^ ^]^ Unlike traditional linear RNA, circRNA can exert its biological functions in diverse ways, including binding to proteins, acting as a microRNA (miRNA) sponge, and encoding peptides.^[^ ^5^, ^6^, ^7^ ^]^ These versatile functions position circRNA as a promising candidate for diagnostic biomarkers and therapeutic targets in various diseases. Indeed, a growing body of evidence has highlighted the close association between circRNA and HCC,^[^ ^8^, ^9^ ^]^ demonstrating that circRNA can regulate HCC tumorigenesis and progression by binding to proteins and modulating their expression and localization.^[^ ^7^, ^10^, ^11^ ^]^ For example, circSTX6 has been shown to promote HCC cell proliferation, migration, and development by binding to HNRNPD, mediating the degradation of ATF3 mRNA, and encoding the circSTX6‐144aa peptide via IRES1‐driven translation.^[^ ^12^ ^]^ Similarly, circLARP1B binds to HNRNPD in the cytoplasm, disrupting its interaction with LKB1 mRNA and thereby reducing LKB1 mRNA and protein levels, which in turn promotes HCC metastasis and lipid accumulation.^[^ ^13^ ^]^ Given these advantages, circRNA holds significant potential as a new target for HCC prevention and diagnosis. Besides, unlike linear mRNA, which is often unstable, inefficient, and immunogenic, circRNA is characterized by its low immunogenicity, cost‐effectiveness, and long half‐life.^[^ ^14^, ^15^, ^16^ ^]^ However, despite these promising features, the molecular and biological functions of circRNA in HCC remain largely unexplored.^[^ ^17^ ^]^

In this study, we identified a novel circRNA, hsa_circ_0006984, formed from the fifth exon of the zinc finger protein 79 (ZNF79) mRNA. Following the nomenclature proposed by Chen et al.^[^ ^18^ ^]^ we renamed it circZNF79(5). Our analysis revealed that circZNF79(5) is highly expressed in HCC tissues and exosomes, as evidenced by data from the GEO database (GSE166678^[^ ^19^ ^]^) and exoRBase 2.0. Intriguingly, despite its high expression, the regulatory role and mechanisms of circZNF79(5) in HCC have not been previously reported. Thus, elucidating its function and mechanisms in HCC progression represents a highly innovative and valuable area of research. Our findings demonstrate that silencing circZNF79(5) significantly inhibits HCC cell proliferation, migration, invasion, and tumor growth both in vitro and in vivo, whereas circZNF79(5) overexpression had the opposite role on HCC cell proliferation and migration. Mechanistic studies revealed that silencing circZNF79(5) promotes p62‐mediated selective autophagic degradation of YBX1 via the AMPK/mTOR signaling pathway. Additionally, circZNF79(5) stabilizes YBX1 protein by recruiting BRCC36 to remove K63‐linked ubiquitin chains from YBX1, thereby promoting HCC progression through the HIF‐1 signaling pathway. Furthermore, we observed a positive correlation between the expression levels of circZNF79(5), YBX1, and BRCC36 in HCC tissues. Collectively, our work highlights the critical role of the oncogenic circZNF79(5)‐YBX1‐BRCC36 axis in HCC progression and identifies it as a potential therapeutic target.

## 2 Results

### 2.1 circZNF79(5) is Upregulated in HCC and Correlated with Poor Prognosis

**Figure 1:**
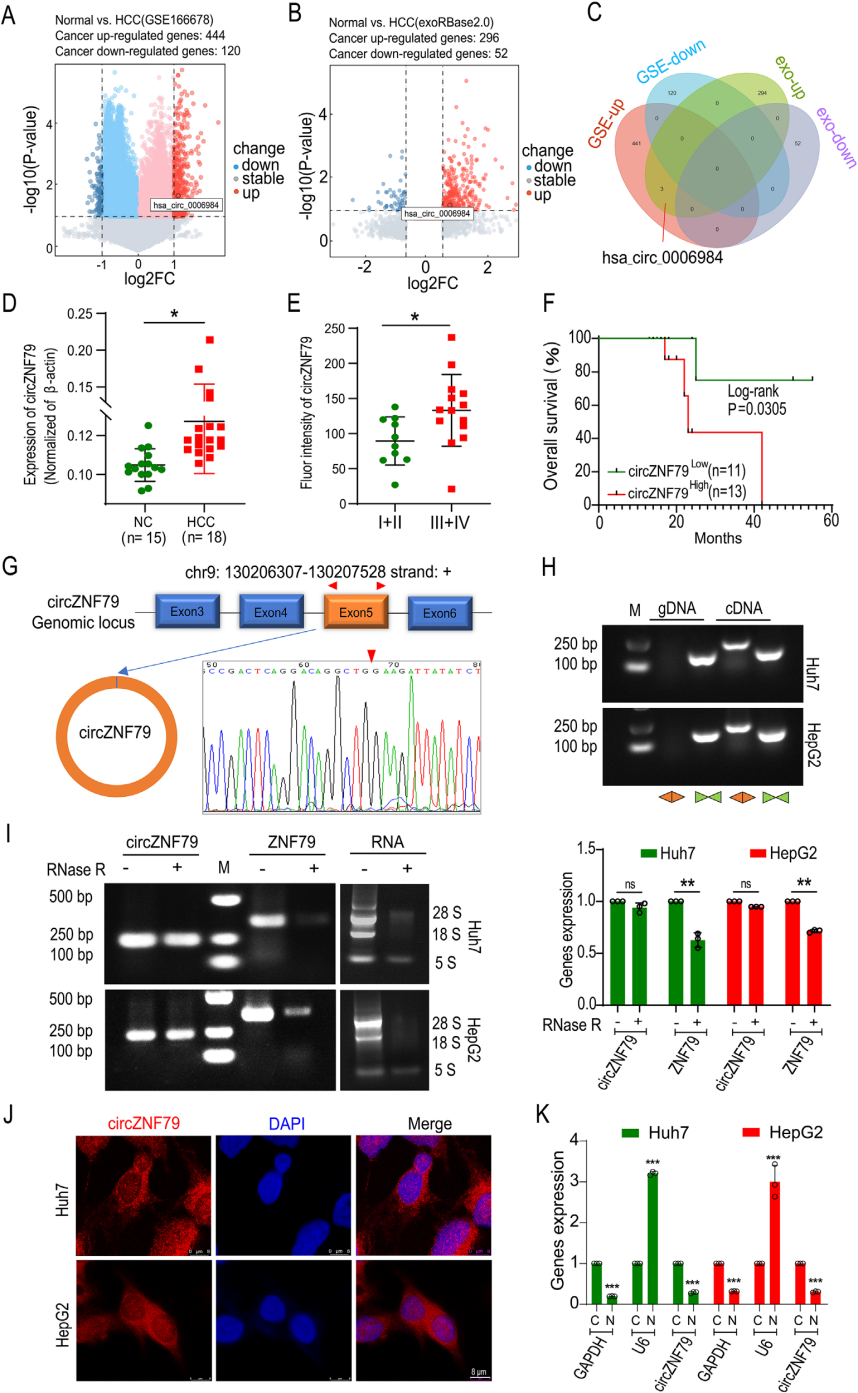
circZNF79(5) is upregulated in HCC tissues and cells. A,B) Volcano plots of differentially expressed genes (DEGs) from GSE166678 (A) and exoRBase2.0 (B). Red dots indicate upregulated genes, while blue dots indicate downregulated genes (*P* < 0.05 and fold change (FC) ≥ 2). circZNF79(5) is highlighted. C) Venn diagram showing the overlap of DEGs identified in GSE166678 and exoRBase2.0. D) Expression levels of circZNF79(5) in HCC tissues detected by qRT‐PCR. E) FISH assays for circZNF79(5) in a tissue microarray of 24 HCC samples, with fluorescence intensity analysis revealing higher expression in advanced tumor stages (Stage III‐IV). F) Kaplan–Meier survival analysis of 24 HCC samples divided into high and low expression groups based on circZNF79(5) fluorescence intensity, showing overall survival rates. G) Sanger sequencing confirmed that circZNF79(5) is generated by back‐splicing of exon 5 of the *ZNF79* gene. H) Presence of circZNF79(5) validated in Huh7 and HepG2 cells using divergent primers in cDNA but not in gDNA. I) qRT‐PCR and DNA electrophoresis demonstrating that circZNF79(5) is resistant to RNase R degradation in Huh7 and HepG2 cells. J) FISH imaging showing that circZNF79(5) is predominantly localized in the cytosolic (Scale bar = 8 µm). K) qRT‐PCR assays after nucleocytoplasmic separation indicate that circZNF79(5) is mainly distributed in the cytoplasm, with GAPDH as a cytoplasmic (C) and U6 a nuclear (N) reference in Huh7 and HepG2 cells. Three independent experiments with three technical replicates were performed. Statistical analysis was conducted using a two‐tailed *t*‐test. ^*^ *P* < 0.05, ^**^ *P* < 0.01, ^***^ *P* < 0.001, *P* > 0.05 (not significant, ns), *n* = 3. Data are presented as mean ± S.E.M.

Emerging evidence highlights the involvement of circRNAs in various cancer‐related processes. To identify circRNAs associated with hepatocellular carcinoma (HCC), we analyzed two independent circRNA microarray datasets (GSE166678 and exoRBase2.0). In GSE166678, we identified 444 upregulated and 120 downregulated circRNAs in tumor tissues compared to adjacent normal tissues (|log2 FC| ≥ 1 and *P* < 0.05, **Figure** **1**A). Similarly, in exoRBase2.0, we found 296 upregulated and 52 downregulated circRNAs (Figure 1B). Notably, three circRNAs were consistently upregulated in both datasets, including hsa_circ_0006984, a novel circRNA with unknown regulatory role and mechanisms reported in all diseases (Figure 1C). We renamed hsa_circ_0006984 as circZNF79(5) (circZNF79 in figures) based on its origin from the back‐splicing of the fifth exon of the *ZNF79* gene, as confirmed by the UCSC Genome Browser. Using divergent primers, we validated the upregulation of circZNF79(5) in clinical HCC samples by qRT‐PCR (Figure 1D). Further, we examined circZNF79(5) expression in a tissue microarray containing 24 HCC samples using FISH. The fluorescence intensity analysis revealed that circZNF79(5) was significantly upregulated in HCC tissues (Figure S1A, Supporting Information, with six examples shown randomly in Figure S1B, Supporting Information), and associated with advanced tumor stages (Stage III‐IV, Figure 1E) and poor prognosis (Figure 1F). Additionally, we analyzed the correlation between circZNF79(5) expression and other clinical features of HCC patients (Supplementary Table 1). Moreover, we next evaluated exosomal circZNF79(5) expressions in the serum of 7 pairs HCC and non‐HCC patients and culture media of Huh7 and HepG2 cells. The exosomes were isolated and identified in Figure S1C–E (Supporting Information). qRT‐PCR showed that exosomal circZNF79(5) expression was overexpressed in HCC patients (Figure S1F, Supporting Information) and HCC cells (Figure S1G, Supporting Information). Likewise in HCC cells, including Huh7, HepG2, Bel‐7402 and MHCC‐97H cells (Figure S1H, Supporting Information), indicating circZNF79(5) might play a central role in HCC.

Sanger sequencing confirmed the full‐length sequence of circZNF79(5) in Huh7 cells, which matched the back‐splicing junction site reported in CircBase (Figure 1G). To verify the circular nature of circZNF79(5), we designed convergent and divergent primers to amplify circZNF79(5) from cDNA and genomic DNA (gDNA) by qRT‐PCR Gel electrophoresis showed that circZNF79(5) could be amplified by both types of primers in cDNA but only by convergent primers in gDNA (Figure 1H). The half‐life of circZNF79(5) was significantly prolonged compared with that of linear ZNF79 mRNA after Actinomycin D (ActD) treatment (Figure S1I, Supporting Information). Furthermore, treatment of the extracted total RNA from Huh7 and HepG2 cells with RNase R, a linear RNA degrader, demonstrated that circZNF79(5) was more resistant to degradation than its linear counterpart, ZNF79 mRNA (Figure 1I). Finally, FISH coupled with cytosolic/nuclear fractionation experiments revealed that circZNF79(5) was predominantly localized in the cytosolic of HCC cells (Figure 1J,K), which was consistent with the CSCD (http://gb.whu.edu.cn/CSCD/#) database. Collectively, these data demonstrate that circZNF79(5) is highly expressed in HCC tissues, cells, and the serum exosomes, and is primarily localized in the cytosolic, suggesting its potential as a biomarker and therapeutic target in HCC.

### 2.2 circZNF79(5) Plays an Oncogenic Role in HCC Cells

**Figure 2:**
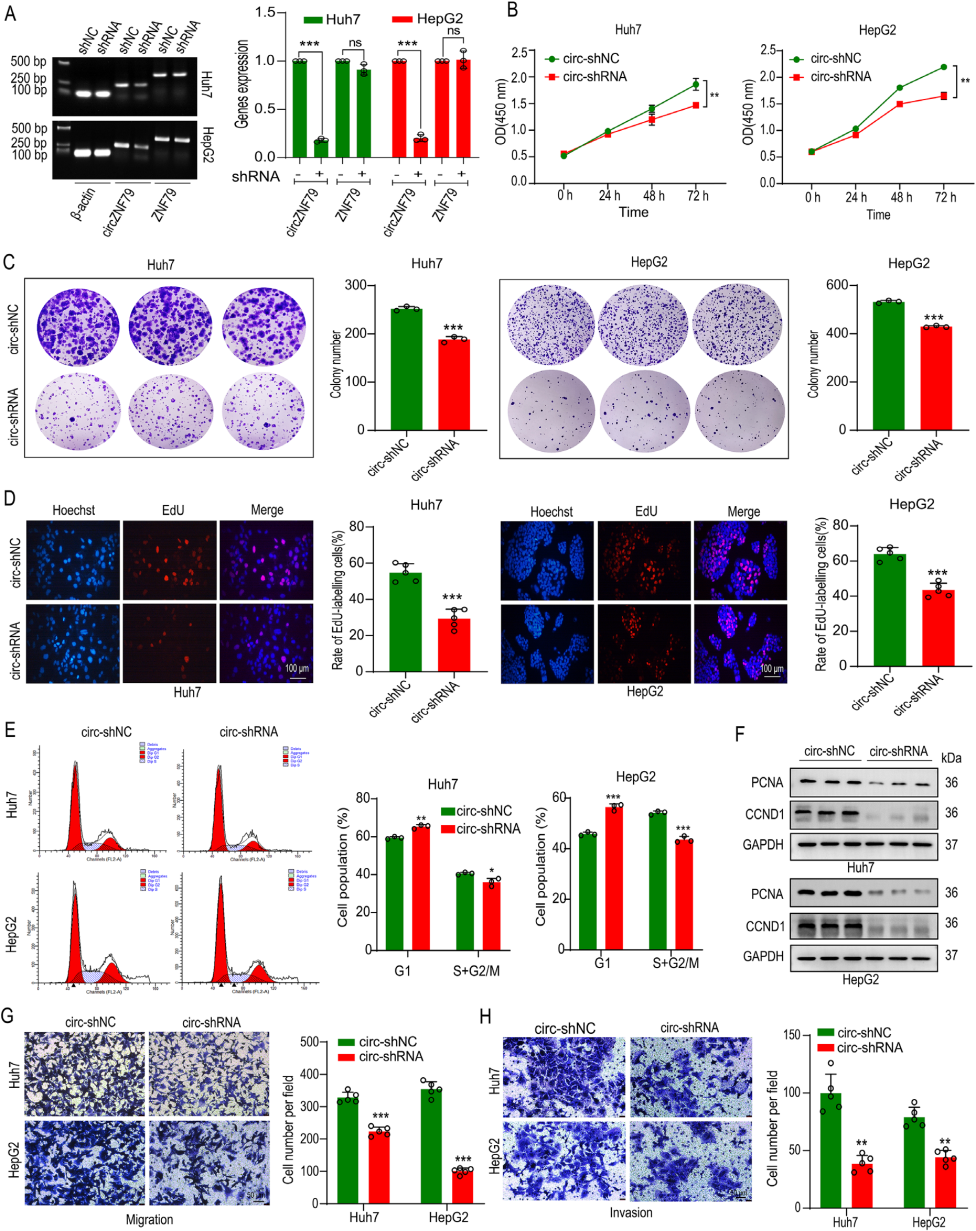
Knockdown of circZNF79(5) inhibits HCC cell proliferation, migration, and invasion in vitro. A) qRT‐PCR validation of circZNF79(5) knockdown, without affecting ZNF79 mRNA, in Huh7 and HepG2 cells transfected with back‐splice junction‐specific shRNA (*n* = 3), the control group underwent normalization processing when been quantified. B–D) CCK8 (B, *n* = 5), colony formation (C, *n* = 3), and EdU assays (D, *n* = 5, Scale bar = 100 µm) demonstrate the inhibitory effects of circZNF79(5) knockdown on cell proliferation in Huh7 and HepG2 cells. E,F) Flow cytometry (E), and WB (F) demonstrate the inhibitory effects of circZNF79(5) knockdown on cell cycle in Huh7 and HepG2 cells. (G, H) Transwell assays show that circZNF79(5) knockdown reduces cell migration G) and matrigel invasion H) in Huh7 and HepG2 cells (*n* = 5, Scale bar = 50 µm). Three independent experiments with three technical replicates were performed. Statistical analysis was conducted using a two‐tailed *t*‐test. ^*^ *P* < 0.01, ^**^ *P* < 0.001, ^***^ *P* < 0.001, and *P* > 0.05 (not significant, ns). Data are presented as mean ± S.E.M.

To elucidate the role of circZNF79(5) in HCC cells, we synthesized and transfected two junction‐specific siRNAs targeting circZNF79(5) (Figure S2A, Supporting Information) into Huh7 cells. The siRNA‐1 more effectively downregulated circZNF79(5) compared to siRNA‐2, without affecting the expression of linear ZNF79 mRNA (Figure S2B, Supporting Information). Additionally, siRNA‐1 more significantly inhibited HCC cell proliferation (Figure S2C,D, Supporting Information). To establish stable knockdown cell lines, we used shRNA targeting circZNF79(5) (based on siRNA‐1) in Huh7 and HepG2 cells. Successful establishment of these knockdown systems was confirmed (**Figure** **2**A). And the overexpression systems were successfully established by using LV5(EF‐1a/GFP&Puro) vector in highly infectious Huh7 and MHCC‐97H cells (Figure S3A,B, Supporting Information). Subsequent assays revealed that silencing circZNF79(5) significantly impeded cell proliferation in both Huh7 and HepG2 cells, as demonstrated by CCK‐8 (Figure 2B) and colony formation assays (Figure 2C). EdU assays (Figure 2D) and cell cycle analysis (Figure 2E,F) further confirmed the pro‐proliferative effects of circZNF79(5). Moreover, Transwell assays showed that circZNF79(5) knockdown markedly inhibited cell migration and invasion abilities (Figure 2G,H), but promoted cell apoptosis (Figure S2E, Supporting Information). Similarly, circZNF79(5) overexpression supported the same conclusion (Figure S3C–E, Supporting Information). Collectively, these findings suggest that circZNF79(5) functions as an oncogene in HCC cells in vitro.

### 2.3 circZNF79(5) Interacts with YBX1

**Figure 3:**
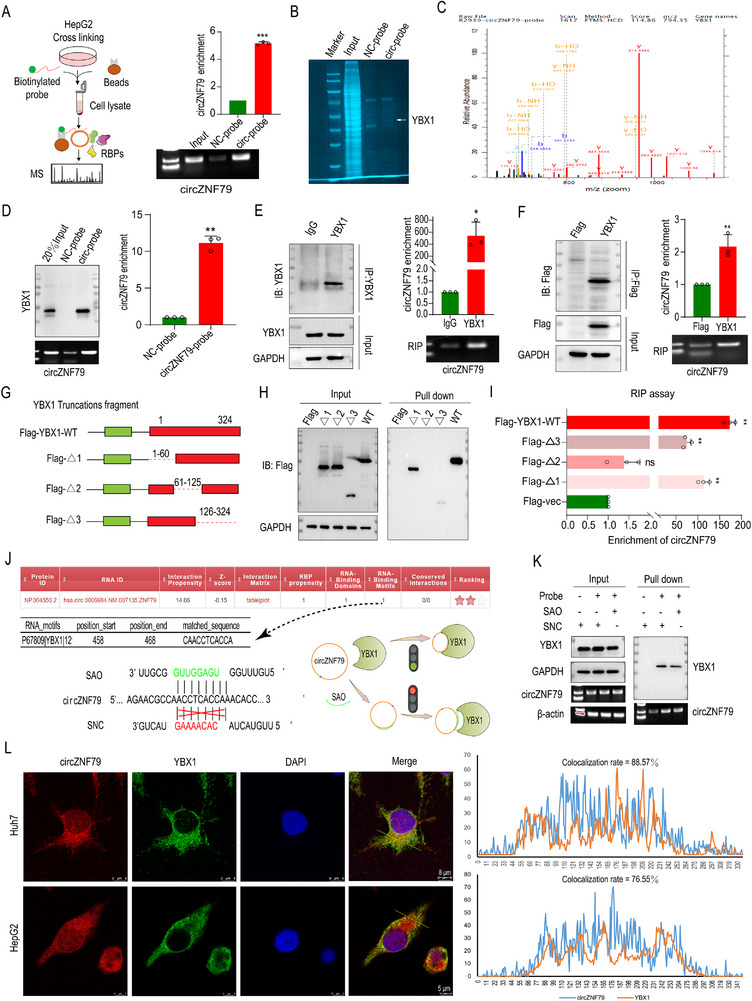
circZNF79(5) Binds to YBX1 Protein. A) Schematic of circZNF79(5) pull‐down assays. B) Coomassie brilliant blue staining after circZNF79(5) pull‐down assays. C) MS identification of YBX1. D) Biotinylated probe pull‐down assay for YBX1 using WB and qRT‐PCR analysis for circZNF79(5) in HepG2 cells. E) Reciprocal RIP assays using YBX1 antibody for circZNF79(5) in HepG2 cells. F) HepG2 cells transfected with pCDNA3.1‐3×FLAG‐YBX1. Reverse RNA RIP assays of circZNF79(5) using Flag beads. G) Diagram of Flag‐tagged WT or truncated mutant plasmids of YBX1. H) HepG2 cells transfected with Flag vector or WT or truncated mutant plasmids of YBX1. RNA pull‐down using a circZNF79(5) probe. WB detected the pull‐downs by anti‐Flag. I) RIP assay showing that the fragment of YBX1 without 61‐125aa loses binding capacity with circZNF79(5). J) Predicted binding sites of circZNF79(5) and YBX1 (catRAPID graph) and design of SAO/SNC targeting this site. K) SAO or SNC transfected into Huh7 cells. The binding sequence of circZNF79(5) for YBX1 was identified by pull‐down assay. L) FISH for circZNF79(5) (red), IF for YBX1 (green), nuclei staining (blue), and merged (yellow) images in Huh7 and HepG2 cells. The merged panel shows colocalization of circZNF79(5) and YBX1, and is quantified. Scale bar = 8/5 µm. Three independent experiments with three technical replicates were performed. Statistical analysis was conducted using a two‐tailed *t*‐test. ^*^ *P* < 0.05, ^**^ *P* < 0.01, and *P* > 0.05 (not significant, ns), *n* = 3. Data are presented as mean ± S.E.M.

Recent studies have highlighted that circRNA‐protein interactions play critical roles in various diseases.^[^ ^7^ ^]^ To identify the proteins that bind to circZNF79(5) and mediate its effects in HCC, we performed RNA pull‐down assays using a biotin‐labeled circZNF79(5) probe. The results showed that circZNF79(5) was successfully enriched compared to the control probe, demonstrating the specificity of our probe (**Figure** **3**A). SDS‐PAGE and Coomassie brilliant blue staining revealed a prominent protein band between 40 and 55 kDa in the circZNF79(5) probe group (Figure 3B). LC‐MS/MS analysis identified YBX1 (50 kDa) as a protein enriched in the circZNF79(5) probe group (Figure 3C). WB of the pulled‐down proteins further confirmed the specific binding of circZNF79(5) to YBX1 (Figure 3D). We validated this interaction using endogenous RIP assays with anti‐YBX1 antibodies (Figure 3E) and exogenous RIP assays with Flag‐Magnetic beads in HepG2 cells overexpressing Flag‐YBX1 (Figure 3F). Furthermore, RIP assay was used to explore whether circZNF79(5) could bind to AGO2, and bioinformatics was used to analyze its competing endogenous RNA (ceRNA) and coding potential. It showed that circZNF79(5) could bind to AGO2 (Figure S4, Supporting Information). Besides, circZNF79(5) showed 284 potential targeted miRNAs and 0.8811 “Coding‐Prob” in circBank (http://www.circbank.cn/#/browse/details?type=human&id=hsa_ZNF79_0000900), “coding” annotation in circBase (https://www.circbase.org/cgi‐bin/singlerecord.cgi?id=hsa_circ_0006984), and an Open Reading Frame (ORF) in CSCD database, respectively, indicating its ceRNA and protein coding potential.

To pinpoint the binding sites of YBX1 on circZNF79(5), we synthesized FLAG‐tagged wild‐type (WT) YBX1 and truncated mutants: Δ1 (1–60 amino acid deletion, A/P‐rich domain), Δ2 (61–125 aa deletion, cold shock domain, CSD), and Δ3 (126–324 aa deletion, C‐terminal domain) (Figure 3G). Pull‐down assays with the biotin‐labeled circZNF79(5) probe showed that the Δ2 mutant was not enriched, indicating that the CSD region (61–125 aa) of YBX1 is essential for binding to circZNF79(5) (Figure 3H). RIP assays further confirmed that the Δ2 fragment did not interact with circZNF79(5) (Figure 3I).

To identify the specific binding region on circZNF79(5), we used the catRAPID tool to predict the interaction sites and found that YBX1 primarily binds to the 458–468 sequence of circZNF79(5). We predicted circZNF79(5)‐YBX1 binding sites using catRAPID and synthesized single morpholino antisense oligos (SAO) targeting this sequence (Figure 3J) and transfected them into Huh7 cells. RNA pull‐down assays showed that targeting the 458–468 sequence blocked the interaction between circZNF79(5) and YBX1 (Figure 3K). Additionally, FISH and IF assays using confocal microscopy revealed that circZNF79(5) and YBX1 were colocalized predominantly in the cytosolic of Huh7 and HepG2 cells with 88.57% and 76.55% colocalization rate, respectively (Figure 3L). Collectively, these results confirm a specific interaction between circZNF79(5) and YBX1, mediated by the CSD region of YBX1 and the 458–468 sequence of circZNF79(5).

### 2.4 YBX1 Mediates circZNF79(5) Oncogenic Function via the HIF‐1 Signaling Pathway

**Figure 4:**
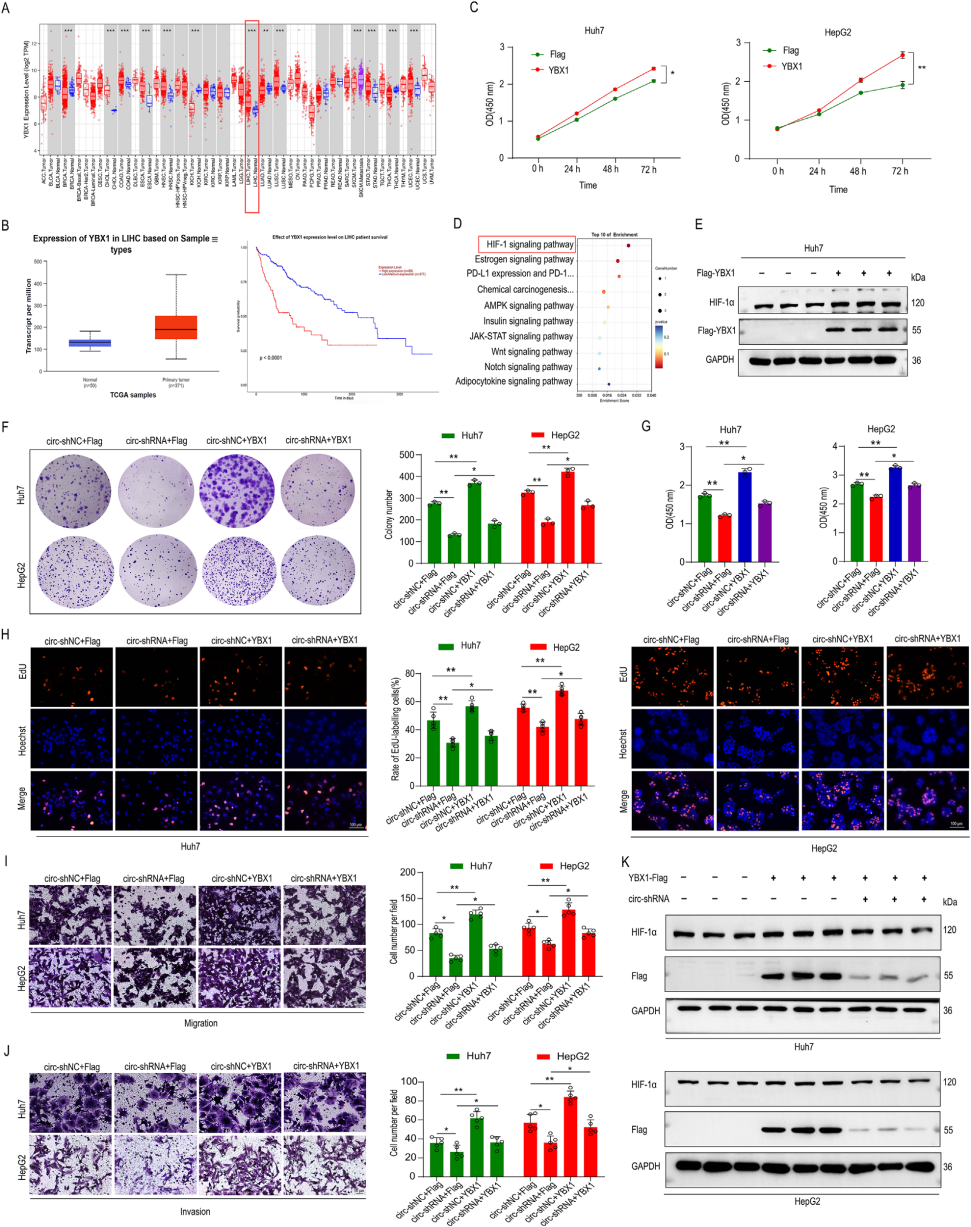
YBX1 Overexpression Reverses the Phenotypic Effects of circZNF79(5) Silencing. A) YBX1 expression in TCGA HCC tumor and normal liver tissues (TIMER: https://cistrome.shinyapps.io/timer/). B) Survival analysis showing worse overall survival in patients with higher YBX1 levels compared to those with lower YBX1 levels (UALCAN: https://ualcan.path.uab.edu/analysis.html). C) CCK8 assay of HCC cells transfected with Flag‐YBX1 or Flag plasmids (*n* = 5). D) Label‐free and KEGG enrichment analysis of differentially expressed proteins (DEPs) in HepG2 cells transfected with circ‐shNC or circ‐shRNA. E) WB analysis of YBX1, HIF‐1α, and GAPDH in Huh7 cells transfected with YBX1‐Flag or Flag‐vector (*n* = 3). F–H) Functional restoration experiments: YBX1 overexpression reversed circZNF79(5) knockdown‐induced proliferative suppression in Huh7 and HepG2 cells, as shown by colony formation (F, *n* = 3), CCK8 assay at 72 h (G, *n* = 5), and EdU assay (H, *n* = 5, Scale bar = 100 µm). I,J) Functional restoration experiments: YBX1 overexpression restored cell migration (I) and invasion (J) phenotypes in Huh7 and HepG2 cells, as shown by Transwell assays (*n* = 5, Scale bar = 50 µm). K) WB analysis of YBX1, HIF‐1α, and GAPDH in Huh7 cells transfected with overexpressing Flag‐YBX1 and circ‐shRNA. Three independent experiments with three technical replicates were performed. Statistical analysis was conducted using a two‐tailed *t*‐test. ^*^ *P* < 0.05, ^**^ *P* < 0.01, and *P* > 0.05 (not significant, ns), *n* = 3. Data are presented as mean ± S.E.M.

YBX1 is a well‐established oncogene.^[^ ^20^ ^]^ Analysis of data from The Cancer Genome Atlas (TCGA) revealed elevated YBX1 levels in HCC tumors compared to normal liver tissues (**Figure** **4**A), correlating with poor prognosis (Figure 4B). To further explore the oncogenic role of YBX1, we overexpressed YBX1 using a Flag‐tagged vector. CCK‐8 (Figure 4C) and EdU assays (Figure S5A, Supporting Information) indicated that YBX1 overexpression enhanced cell proliferation in Huh7 and HepG2 cells. Wound‐healing assays also showed that YBX1 upregulation significantly promoted cell migration (Figure S5B, Supporting Information). Label‐free quantitative proteomics and KEGG enrichment analysis of HepG2 cells transfected with circ‐shNC and circ‐shRNA revealed significant enrichment of the HIF‐1 signaling pathway (Figure 4D), in which three related proteins of HIF‐1 signaling pathway were downregulated in the circ‐shRNA group. Given that YBX1 has been shown to activate HIF‐1α translation and promote sarcoma metastasis,^[^ ^21^ ^]^ we hypothesized that HIF‐1α is a downstream target of YBX1. Consistent with this, WB demonstrated that YBX1 overexpression significantly increased HIF‐1α protein levels in Huh7 (Figure 4E, quantified Figure S9B, Supporting Information) and HepG2 cells (Figure S5C, quantified Figure S9C, Supporting Information).

To determine whether circZNF79(5) exerts its oncogenic effects via YBX1, we conducted functional restoration experiments. Colony formation, CCK‐8, EdU, Transwell, and invasion assays showed that circZNF79(5) knockdown inhibited cell proliferation, migration, and invasion in Huh7 and HepG2 cells. These inhibitory effects were reversed by YBX1 overexpression (Figure 4F–J). Conversely, circZNF79(5) overexpression promoted cell proliferation and migration in Huh7 and MHCC‐97H cells, and these promoting effects were reversed by YBX1 knockdown (Figure S5D–F, Supporting Information). Additionally, silencing circZNF79(5) decreased YBX1 and HIF‐1α protein levels (Figure 4K, quantified in Figure S9A, Supporting Information). Collectively, these results suggest that circZNF79(5) promotes HCC progression by targeting YBX1 and activating the HIF‐1 signaling pathway.

### 2.5 Silencing circZNF79(5) Promotes Selective Autophagic Degradation of YBX1 via the AMPK/mTOR Signaling Pathway

**Figure 5:**
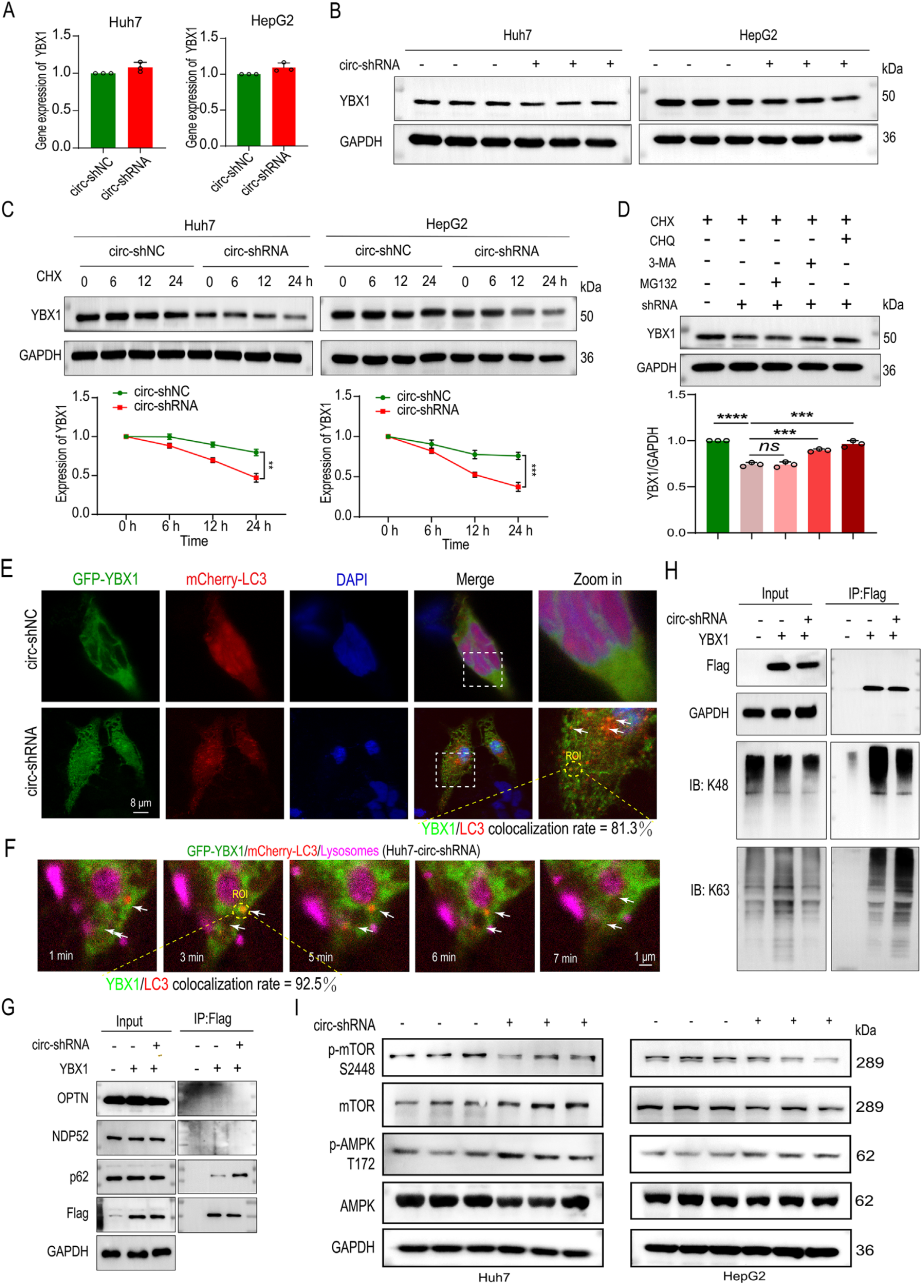
Silencing circZNF79(5) Promotes Selective Autophagic Degradation of YBX1 via the AMPK/mTOR Signaling Pathway. A,B) qRT‐PCR analysis of YBX1 mRNA (A) and WB analysis of YBX1 and GAPDH proteins (B) in HCC cells transfected with circ‐shNC or ‐shRNA. C) WB analysis of YBX1 and GAPDH in HCC cells transfected with circ‐shNC or ‐shRNA and treated with CHX (200 µg/mL) at 0, 6, 12, and 24 h. D) WB analysis of YBX1 stabilization in Huh7 cells transfected with circ‐shNC or ‐shRNA, followed by treatment with DMSO, MG132 (20 µm), 3‐MA (50 µm), or CHQ (20 µm) for 6 h. E) Huh7 cells co‐transfected with GFP‐YBX1 and LC3‐mCherry and treated with RAPA (20 nm) for 6 h. Confocal microscopy (STED) analysis of GFP‐YBX1 and LC3‐mCherry colocalization and quantified (Scale bar = 8 µm). F) Live‐cell imaging of YBX1‐GFP and LC3‐mCherry colocalization and quantified in Huh7 cells. G,H) Huh7 cells transfected with Flag‐YBX1. Co‐IP and WB analysis verified the interaction of YBX1 with p62, but not other cargo receptors (G), and showed increased K63‐linked ubiquitination of YBX1, but not K48 (H). I) WB analysis of p‐mTOR, mTOR, p‐AMPK, AMPK, and GAPDH in Huh7 cells transfected with circ‐shNC or ‐shRNA. Numbers indicate phosphorylated amino acid sites. Three independent experiments with three technical replicates were performed. Statistical analysis was conducted using a two‐tailed *t*‐test (*n* = 3). ^**^ *P* < 0.01, ^***^ *P* < 0.001, ^****^ *P* < 0.0001. Data are presented as mean ± S.E.M.

To elucidate the mechanism by which silencing circZNF79(5) decreases YBX1 protein levels, we first confirmed that circZNF79(5) knockdown did not affect YBX1 mRNA levels (**Figure** **5**A,B, quantified Figure S9D, Supporting Information). Inversely, circZNF79(5) overexpression increased YBX1 protein levels (Figure S6A, Supporting Information). But disrupting the interaction between YBX1 and circZNF79(5) using SAO also decreased YBX1 protein levels (Figure S6B,C, quantified Figure S9E, Supporting Information). This suggested that circZNF79(5) regulates YBX1 post‐transcriptionally. We then hypothesized that circZNF79(5) stabilizes YBX1. Treatment with cycloheximide (CHX) revealed that YBX1 protein stability was significantly reduced upon circZNF79(5) silencing (Figure 5C) or upon transfected with SAO (Figure S6D, Supporting Information), but increased upon circZNF79(5) overexpression (Figure S6E, Supporting Information). To determine the degradation pathway involved, we used various inhibitors. The autophagy inhibitors 3‐MA and CHQ significantly increased the effect of circZNF79(5) silencing on YBX1 levels, while the proteasome inhibitor MG132 had no effect (Figures 5D and S6F, quantified Figure S9F, Supporting Information). This indicated that circZNF79(5) silencing promotes YBX1 degradation via autophagy rather than the proteasome pathway. Live‐cell imaging with LC3‐mCherry further demonstrated that YBX1‐GFP colocalized with autophagosomes and degraded in lysosomes upon circZNF79(5) silencing with 81.3% and 92.5% colocalization rate in the region of interest (ROI), respectively (Figure 5E,F, and Video S1, Supporting Information).

We further explored the role of selective autophagy in YBX1 degradation. Co‐IP and WB revealed that YBX1 interacts with p62, a key cargo receptor for selective autophagy (Figure 5G). Interestingly, YBX1 bound more p62 in circ‐shRNA cells than in circ‐shNC cells, suggesting that circZNF79(5) silencing enhances the interaction between YBX1 and p62. To further verify that p62 is a selective autophagy receptor for YBX1, we synthesized and screened the siRNAs of p62 (Figure S6G, Supporting Information). Furthermore, silencing p62 reversed YBX1 down‐expression upon circZNF79(5) knockdown (Figure S6H, Supporting Information). And CHX assay showed the same result (Figure S6I, Supporting Information). Additionally, WB showed increased LC3‐II and decreased p62 levels in circ‐shRNA cells, indicating activated autophagy (Figure S6J, Supporting Information). And the autophagy flux formation showed increased LC3‐mCherry‐GFP dot accumulation upon circZNF79(5) silencing in HCC cells transfected with LC3‐mCherry‐GFP (Figure S6K, Supporting Information). The K48 ubiquitin chain is considered to be a typical signal for proteasome degradation of target proteins, while the K63 ubiquitin chain can be degraded by autophagy, targeting substrates.^[^ ^22^ ^]^ To investigate the role of ubiquitin chains in YBX1 degradation, we transfected Huh7‐shNC and ‐shRNA cells with Flag or Flag‐YBX1. Co‐IP and WB revealed increased K63‐linked ubiquitination of YBX1 upon circZNF79(5) silencing, but not K48‐linked ubiquitination (Figure 5H). This indicated that circZNF79(5) stabilizes YBX1 by preventing K63‐linked ubiquitin‐dependent selective autophagic degradation.

Finally, we explored the signaling pathway involved in circZNF79(5)‐mediated autophagy. Label‐free quantitative proteomics identified 293 downregulated and 168 upregulated proteins in circ‐shRNA‐transfected HepG2 cells compared to circ‐shNC cells (Figure S6L, Supporting Information), which were involved in RNA binding, protein binding, etc. (Figure S6M, Supporting Information). Bioinformatics analysis highlighted the AMPK signaling pathway (Figure S6N, Supporting Information), which is known to regulate autophagy.^[^ ^23^ ^]^ WB confirmed that circZNF79(5) silencing increased p‐AMPK (T172) and decreased p‐mTOR (S2448) levels in Huh7 and HepG2 cells (Figure 5I). Collectively, these results demonstrate that silencing circZNF79(5) promotes selective autophagic degradation of YBX1 via the AMPK/mTOR signaling pathway.

### 2.6 circZNF79(5) Recruits BRCC36 to Stabilize YBX1 by Removing K63‐Linked Ubiquitin

**Figure 6:**
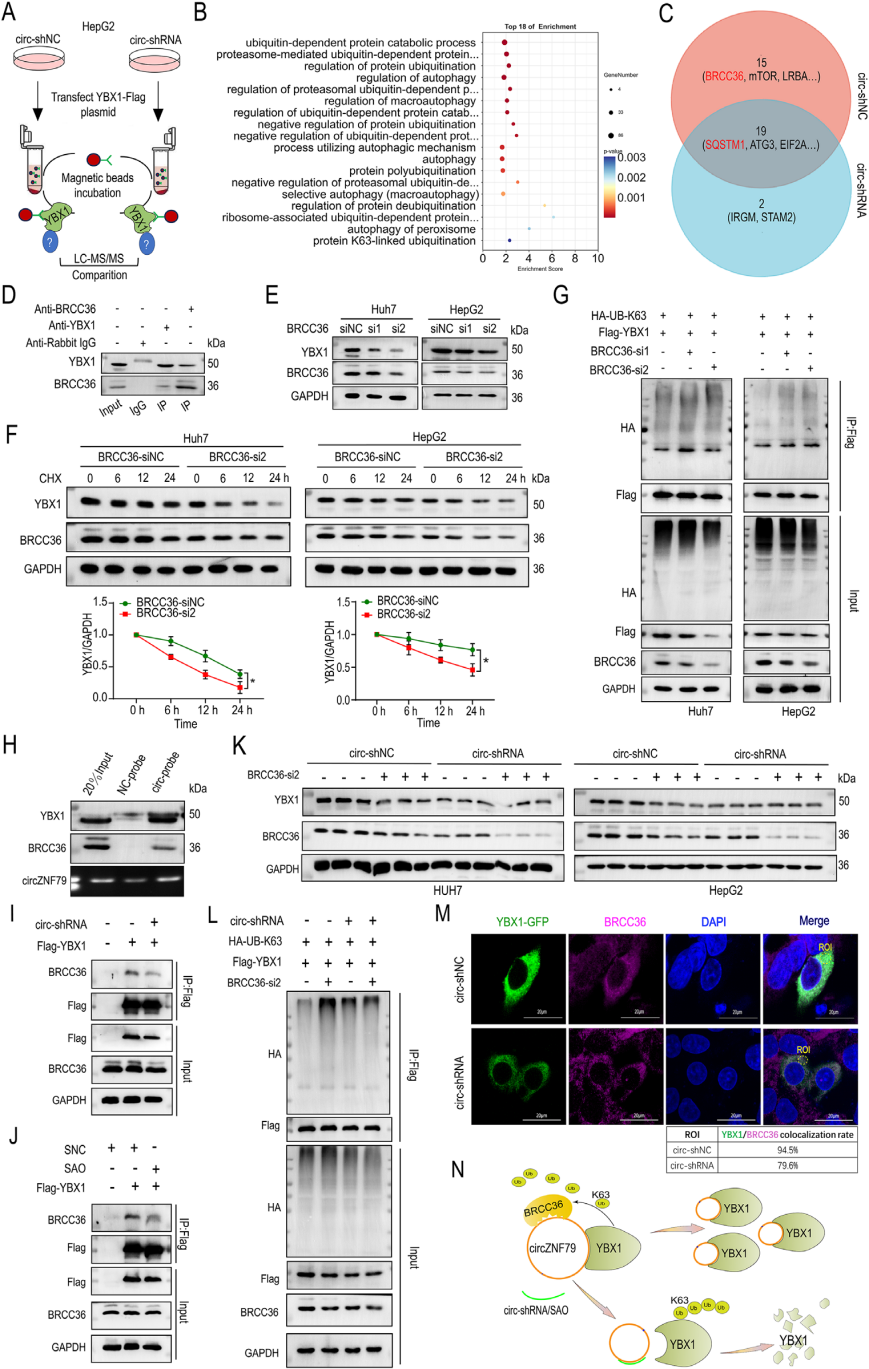
circZNF79(5) Recruits BRCC36 to Stabilize YBX1 by Removing K63‐Linked Ubiquitin. A) Schematic of MS analysis of YBX1 immunoprecipitation in HepG2 cells transfected with circ‐shRNA or ‐shNC. B,C) GO (B) and Venn analysis (C) of DEPs in YBX1‐immunoprecipitated complexes involved in ubiquitination and autophagy from HepG2 cells transfected with Flag‐YBX1 and either circ‐shNC or ‐shRNA. D) Co‐IP assay verifying the interaction between BRCC36 and YBX1. E) Effects of BRCC36 knockdown by two siRNAs on YBX1 expression. F) WB analysis of YBX1 in Huh7 and HepG2 cells transfected with BRCC36‐si2 or ‐siNC and treated with CHX (200 µg mL^−1^). G) Huh7 and HepG2 cells transfected with plasmids expressing Flag‐YBX1, HA‐K63‐linked ubiquitin, and BRCC36‐si2 or ‐siNC. Lysates were subjected to immunoprecipitation, and the polyubiquitination status of YBX1 was analyzed by WB. H) RNA pull‐down assays showing the association of circZNF79(5) with YBX1 and BRCC36. I,J) Exogenous Co‐IP analysis using Flag beads in Huh7 cells transfected with circ‐shRNA or ‐shNC (I) or SAO (J), showing reduced association between BRCC36 and YBX1 upon circZNF79(5) knockdown. K) WB assay measuring YBX1 and BRCC36 expression in Huh7 and HepG2 cells transfected with circ‐shRNA or ‐shNC and BRCC36‐si2 or ‐siNC. L) Huh7 cells transfected with circ‐shRNA or ‐shNC and plasmids expressing Flag‐YBX1, HA‐K63‐linked ubiquitin, and BRCC36‐si2 or ‐siNC. Lysates were subjected to immunoprecipitation, and the polyubiquitination status of YBX1 was analyzed by WB. M) IF analysis of BRCC36 in Huh7 cells transfected with circ‐shRNA or shNC and expressing YBX1‐GFP, and quantified. Scale bars = 20 µm. N) Proposed model: circZNF79(5) recruits BRCC36 to remove K63‐linked ubiquitin from YBX1, stabilizing it. Three independent experiments with three technical replicates were performed. Statistical analysis was conducted using a two‐tailed *t*‐test, ^*^ *P* < 0.05. Data are presented as mean ± S.E.M.

To explore the ubiquitination mechanism and key molecules involved in circZNF79(5)‐mediated YBX1 stabilization, MS was performed on YBX1‐immunoprecipitated complexes from HepG2 cells transfected with Flag‐YBX1 and either circ‐shNC or ‐shRNA (**Figure** **6**A). GO analysis revealed that DEPs bound to YBX1 were closely related to K63‐linked ubiquitination and selective autophagy (Figure 6B). Among these, BRCC36 and SQSTM1 (p62) were identified as potential interactors (Figure 6C). To confirm that circZNF79(5) modulates K63‐linked ubiquitination of YBX1, Huh7 cells were co‐transfected with Flag‐YBX1, HA‐UB‐K48, or HA‐UB‐K63, along with circ‐shRNA or ‐shNC. Co‐IP and WB showed that circZNF79(5) silencing enhanced K63‐linked ubiquitination of YBX1 without affecting K48 linkages (Figure S7A, Supporting Information), but circZNF79(5) overexpression weakened K63‐linked ubiquitination of YBX1 (Figure S7B, Supporting Information), consistent with earlier results on YBX1‐dependent K63 chain ubiquitination and autophagic degradation.

BRCC36, a K63 chain deubiquitinating enzyme (DUB), may stabilize YBX1 by removing K63‐linked ubiquitin chains in HCC cells with high circZNF79(5) expression. Co‐IP assays using anti‐YBX1 and anti‐BRCC36 antibodies confirmed the interaction between YBX1 and BRCC36 (Figure 6D). Silencing BRCC36 with siRNA decreased YBX1 protein levels in Huh7 and HepG2 cells (Figure 6E). CHX chase assays further demonstrated that BRCC36 knockdown accelerated YBX1 degradation in CHX‐treated HCC cells, an effect that could be inhibited by 3‐MA or CHQ (Figure 6F; Figure S7C, Supporting Information). Additionally, silencing BRCC36 increased YBX1 ubiquitination levels (Figure 6G), suggesting that BRCC36 stabilizes YBX1 by deubiquitination. IF microscopy also confirmed the colocalization of YBX1 and BRCC36 (Figure S7D, Supporting Information).

Given that circZNF79(5) is not a DUB, it was hypothesized that circZNF79(5) might function as a platform to recruit BRCC36 to YBX1. Indeed, pull‐down assays showed that both BRCC36 and YBX1 were captured by circZNF79(5), indicating the formation of a circZNF79(5)‐BRCC36‐YBX1 complex (Figure 6H). Silencing circZNF79(5) with shRNA (Figure 6I; Figure S7E, Supporting Information) or disrupting its binding to YBX1 with SAO (Figure 6J; Figure S7F, Supporting Information) significantly reduced the association between BRCC36 and YBX1. Notably, the stabilizing effect of BRCC36 on YBX1 was dependent on circZNF79(5), as evidenced by the attenuated decrease in YBX1 protein levels (Figure 6K, quantified Figure S9G, Supporting Information) and increased K63‐polyubiquitination of YBX1 (Figure 6L) upon circZNF79(5) knockdown. IF microscopy further confirmed that the colocalization of YBX1 and BRCC36 was significantly reduced upon circZNF79(5) knockdown (Figure 6M). Consistent with these findings, silencing circZNF79(5) weakened the inhibitory effects of BRCC36 knockdown on HCC cell proliferation (Figure S8A, Supporting Information) and migration (Figure S8B, Supporting Information). Collectively, these results suggest that circZNF79(5) recruits BRCC36 to cleave K63‐linked polyubiquitin chains on YBX1, thereby protecting it from selective autophagic degradation (Figure 6N).

### 2.7 In Vivo Validation of circZNF79(5) Function in HCC

**Figure 7:**
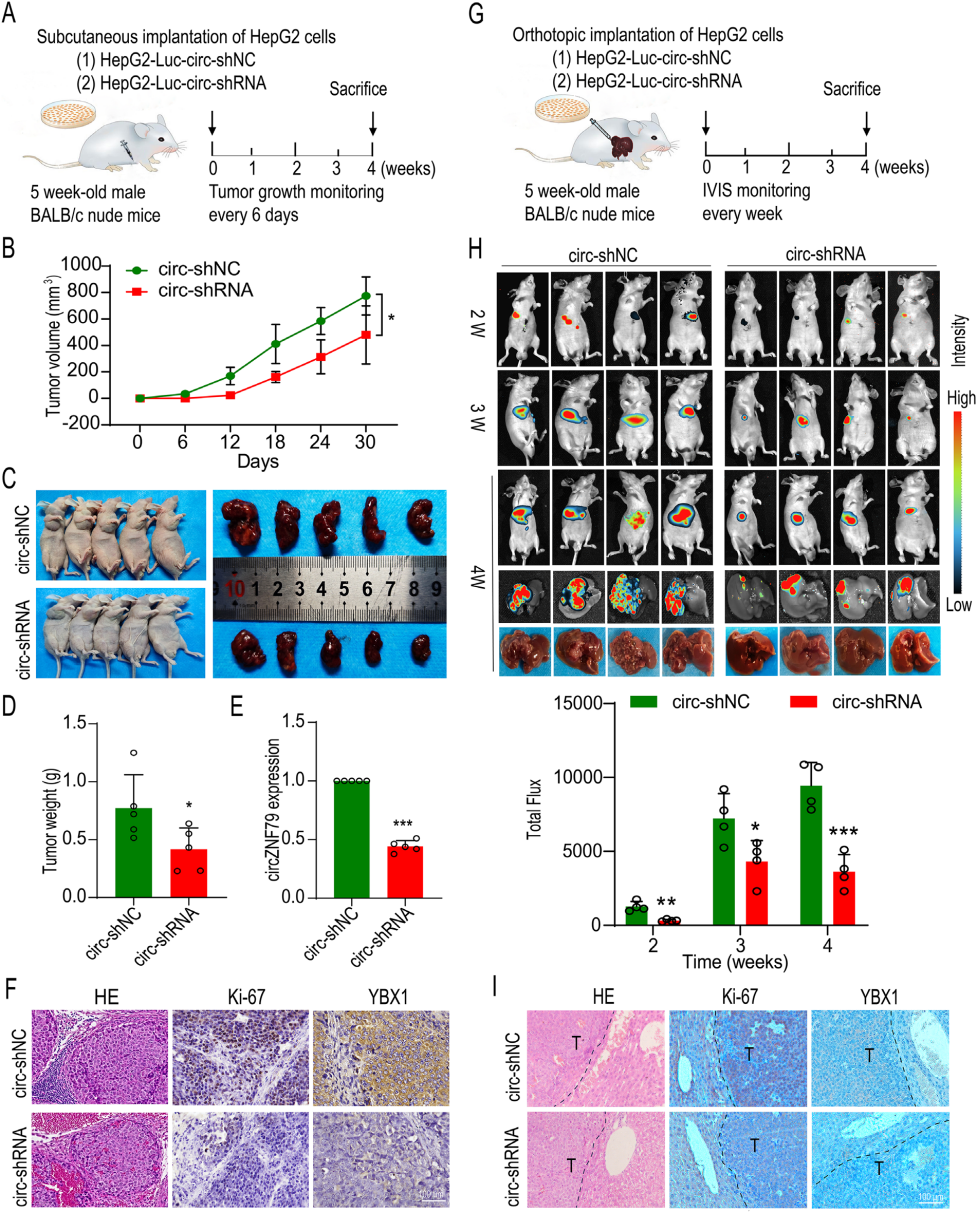
Silencing circZNF79(5) Significantly Inhibits HCC Progression in Vivo. A) Schematic of the subcutaneous tumor model. B,D) Volume (B) and weight (D) of subcutaneous tumors excised from mice at the endpoint (*n* = 5). (C) BALB/c nude mice were subcutaneously injected with HepG2 cells transfected with circ‐shRNA or ‐shNC. Animals were sacrificed at week 4. Appearance of mice (left) and tumors (right) at the endpoint (*n* = 5). E) circZNF79(5) expression in tumors from circ‐shRNA and ‐shNC mice (*n* = 5). F,I) H&E and IHC staining of Ki67 and YBX1. T indicates tumor. Scale bars = 100 µm. G) Schematic of the orthotopic tumor model. HepG2‐Luc cells were transfected with circ‐shRNA or ‐shNC and injected in situ into the liver of male BALB/c nude mice. In vivo luminescent imaging was performed weekly for 4 weeks. Animals were sacrificed at the endpoint. H) IVIS images of orthotopic tumor growth at three time points and ex vivo fluorescence images of isolated livers at the endpoint (*n* = 4). Statistical analysis was performed using a two‐tailed *t*‐test. ^*^ *P* < 0.05, ^**^ *P* < 0.01, and ^***^ *P* < 0.001. Data are presented as mean ± S.E.M.

To further validate the biological function of circZNF79(5) in vivo, subcutaneous tumor models were established using HepG2 cells transfected with circ‐shRNA or ‐shNC (**Figure** **7**A). Tumor volume (Figure 7B) and weight (Figure 7D) were significantly reduced in the circZNF79(5) knockdown group compared to the control (circ‐shNC) group (Figure 7C). The expression of circZNF79(5) in tumor tissues was confirmed by qRT‐PCR (Figure 7E). Histological analysis via H&E and IHC staining revealed decreased expression of Ki‐67 and YBX1 in the circZNF79(5) knockdown group (Figure 7F). Additionally, an orthotopic xenograft model was employed to investigate the effects of circZNF79(5) on tumor growth (Figure 7G). The growth rate of fluorescence intensity was significantly impeded in the circZNF79(5) knockdown group compared to the control group (Figure 7H). Consistent with the subcutaneous model, H&E and IHC staining of Ki‐67 and YBX1 indicated reduced expression in the circZNF79(5) knockdown group (Figure 7I). Collectively, these findings suggest that knockdown of circZNF79(5) inhibits HCC cell proliferation and tumor progression in vivo.

### 2.8 circZNF79(5) Expression is Positively Correlated to YBX1 and BRCC36 in HCC Samples

**Figure 8:**
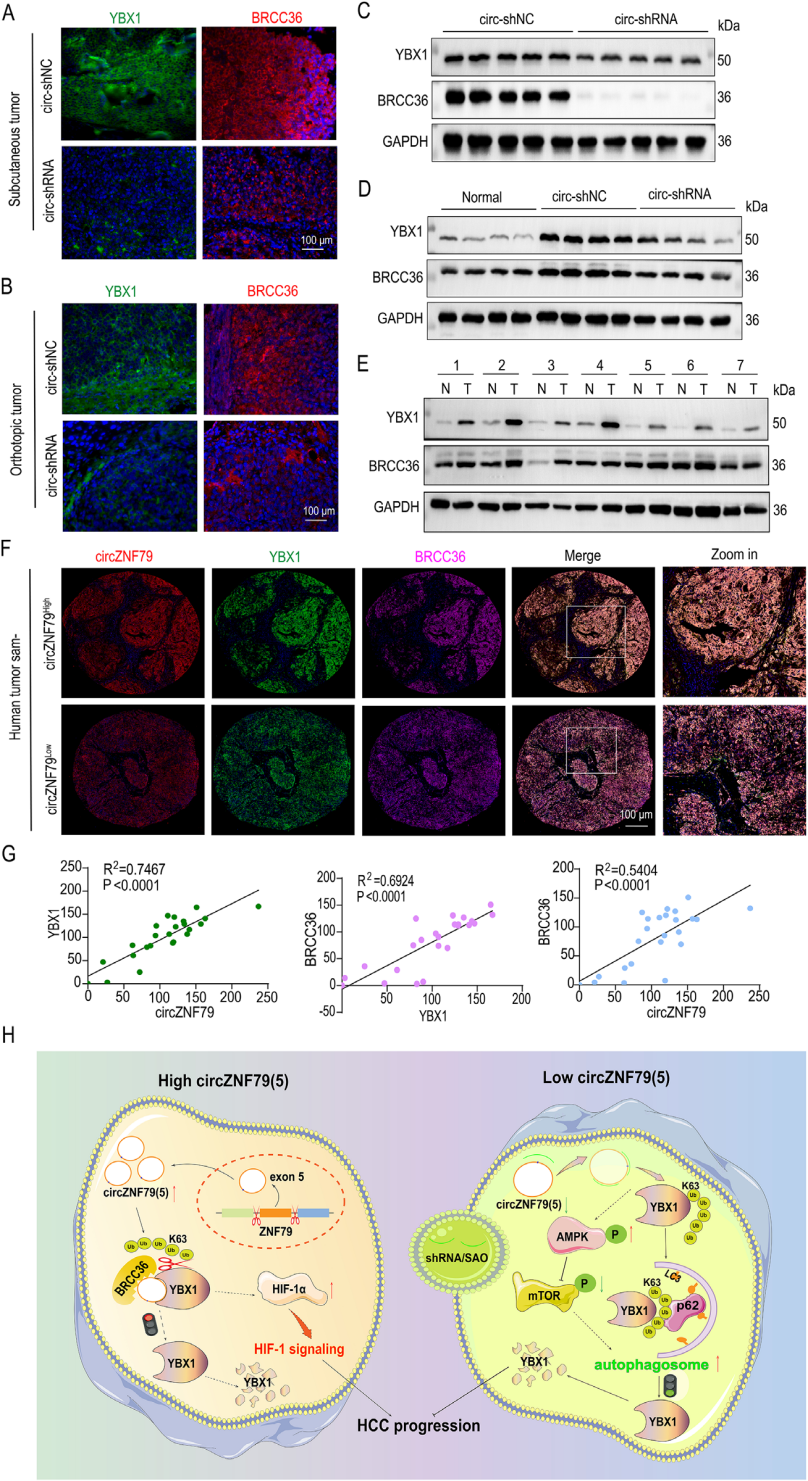
circZNF79(5) Expression Correlates with YBX1 and BRCC36 in HCC Samples. A,B) IF assays measuring YBX1 and BRCC36 expression in subcutaneous (A) and orthotopic (B) tumors from mice. Scale bars = 100 µm. (C, D) WB analysis of YBX1 and BRCC36 expression in subcutaneous C) and orthotopic D) tumors with circZNF79(5) knockdown. E) Representative WB of YBX1 and BRCC36 in 7 matched HCC and normal control (NC) samples. F,G) FISH assays for circZNF79(5) and IF assays for YBX1 and BRCC36 in a tissue microarray of 24 HCC samples (F), with correlation analysis (G). Scale bars = 100 µm. H) Proposed model: In HCC, circZNF79(5) binds to YBX1, recruits BRCC36 to remove K63‐linked ubiquitin chains, and promotes HCC progression via the HIF‐1 signaling pathway. Conversely, circZNF79(5) silencing activates the AMPK/mTOR pathway, inducing p62‐mediated selective autophagic degradation of YBX1.

To further confirm the positive correlation between circZNF79(5), YBX1 and BRCC36, we examined YBX1 and BRCC36 expression by IF in subcutaneous tumor (**Figure** **8**A) and orthotopic tumor (Figure 8B), which showed reduced YBX1 and BRCC36level in keeping with circZNF79(5) decreasing. WB results of subcutaneous tumor (Figure 8C) and orthotopic tumor (Figure 8D) were consistent with IF. In addition, we sought to explore their clinical relevance in tumor samples. WB results of 7 representative HCC samples showed a higher YBX1 and BRCC36 protein level in tumors (Figure 8E). Moreover, we simultaneously examined circZNF79(5) expression by FISH and YBX1, BRCC36 expression by IF in 24 HCC sample tissue array. Uniformly, HCC tissues with higher circZNF79(5) expression exhibited a higher YBX1 and BRCC36 protein level (Figure 8F), and the expression correlation analysis indicated a positively correlated expression of circZNF79(5)‐YBX1‐BRCC36 (Figure 8G). All in all, we found that up‐regulated circZNF79(5) stabilizes proto‐oncogene YBX1 protein by recruiting BRCC36 to remove the K63‐Linked ubiquitination of YBX1 and activates the HIF‐1 signaling pathway to promote HCC progression. Inversely, down‐regulated circZNF79(5) weakens the binding of BRCC36 and YBX1, which activates p62‐mediated selective autophagic degradation of YBX1 via the AMPK/mTOR signaling pathway to inhibit HCC progression (Figure 8H).

## 3 Discussion

The rapid advancement of gene therapy, particularly spurred by the Nobel Prizes in Physiology or Medicine in 2023 and 2024, has accelerated its clinical translation. Among emerging therapeutic targets, circular RNAs (circRNAs) have garnered significant attention due to their unique properties: structural stability, sequence conservation, and tissue‐specific expression. These features make circRNAs promising candidates for gene regulation, biomarkers, and therapeutic targets in various diseases, including hepatocellular carcinoma (HCC).^[^ ^14^, ^17^ ^]^ CircRNAs have been shown to act as protein‐binding RNAs, miRNA sponges, translational templates, and biomarkers, revealing a new dimension in gene regulation.^[^ ^17^ ^]^

At present, there are already many publicly available HCC high‐throughput sequencing and circRNA sequencing databases online. Among them, circRNAs with unknown functions still account for the majority. It is also a common method to screen circRNAs with similar results from more than one database for research.^[^ ^24^, ^25^ ^]^ In this study, we identified circZNF79(5) as a highly expressed circRNA in both HCC tissues and extracellular vesicles, but a novel circRNA with unknown regulatory role and mechanisms reported in all diseases. Its role and mechanisms have not been previously reported in all diseases including HCC. Our primary goal was to elucidate its function and mechanism as a potential diagnostic biomarker and therapeutic target. We confirmed that circZNF79(5) is highly expressed in HCC cell lines and clinical tissues. Regrettably, we had not collected HCC and adjacent tissues to verify its high expression through high‐throughput sequencing. Interestingly, its high expression in HCC cells and patients serum exosomes suggests that circZNF79(5) may act as a secreted factor involved in regulating HCC progression and serve as a potential diagnostic biomarker for HCC. Cellular experiments in vitro, along with subcutaneous and orthotopic tumor models in vivo further demonstrated that circZNF79(5) promotes cell proliferation, migration, invasion, and tumor growth, while inhibits apoptosis, suggesting its oncogenic role in HCC innovatively.

CircRNA‐protein interactions are critical in various diseases.^[^ ^26^, ^27^, ^28^ ^]^ Our study identified YBX1 as a binding partner of circZNF79(5). YBX1 is an oncogenic protein that promotes HCC cell proliferation, apoptosis, autophagy, metastasis, and drug resistance via mediating circRNA‐YBX1 phase separation, regulating signaling pathway, phosphorylation, etc.^[^ ^20^, ^29^, ^30^, ^31^, ^32^, ^33^ ^]^ We found that YBX1 overexpression increases HIF‐1α protein levels and rescues the phenotypes of circZNF79(5) silencing, indicating that YBX1 facilitates HCC progression via the HIF‐1 signaling pathway, which was partially consistent with previous research.^[^ ^30^, ^34^ ^]^ Future work will elucidate how circZNF79(5), through its interaction with YBX1, modulates the HIF‐1 signaling axis to influence HCC progression. Further analysis revealed that circZNF79(5) binds to the 61‐125aa region of YBX1, specifically targeting the 458‐468 sequence of circZNF79(5) for interaction. This provides a theoretical basis for the subsequent preparation of small molecule drugs for HCC by targeting circZNF79(5) and YBX1.

Our investigation into the regulatory mechanisms revealed that circZNF79(5) affects YBX1 protein levels and stability, but not mRNA levels. We first found that YBX1 is degraded via the autophagy‐lysosome pathway upon circZNF79(5) silencing in HCC. Autophagy can be highly selective, with the specificity of substrate recognition by selective autophagy dependent on cargo receptors, including p62 (SQSTM1), NDP52 (CALCOCO2), OPTN, and more.^[^ ^35^ ^]^ The many selective autophagy target cargos are linked to numerous physiological roles and diseases.^[^ ^36^, ^37^ ^]^ We further found YBX1 interacts with p62 via the AMPK/mTOR signaling pathway, promoting its selective autophagy. However, circZNF79(5) stabilizes YBX1 by facilitating its interaction with BRCC36, a K63 chain deubiquitinating enzyme and an oncogene in HCC,^[^ ^38^, ^39^ ^]^ thereby suppressing YBX1 degradation. Furthermore, we confirmed that circZNF79(5) knockdown significantly inhibits HCC progression, with expression levels positively correlating with the above two oncogenic proteins, YBX1 and BRCC36, in clinical samples.

Interestingly, we found circZNF79(5) can bind to AGO2, and bioinformatics analysis forecasted its ceRNA role, indicating that circZNF79(5) may not only function through RBPs, but also through acting as a ceRNA and encoding protein, and may play a role in diseases such as HCC. Our future work will explore the role and functional mechanism by which circZNF79(5) acts as a ceRNA or transcript to regulate disease progression.

Collectively, our findings demonstrated that circZNF79(5) functions as an oncogene in HCC by stabilizing YBX1 through BRCC36‐mediated deubiquitination to promote HCC progression via the HIF‐1 signaling pathway, and preventing YBX1 from p62‐mediated selective autophagy via the AMPK/mTOR pathway. Furthermore, this study highlights the circZNF79(5)‐YBX1‐BRCC36 axis as a potential therapeutic target in HCC.

## 4 Experimental Section

### Construction of HepG2 Cell Line Stably Expressing Firefly Luciferase

To generate a HepG2 cell line stably expressing firefly luciferase, HepG2 cells were transfected with the Luciferase‐mCherry‐Puro lentiviral vector purchased from Hanbio Aden Vector Institute (Shanghai, China). Stable transfectants were selected by serial dilution and confirmed using immunofluorescence (IF) microscopy and the UVP BioSpectrum Advanced In Vivo 695 imaging system (Jena, Germany). Subsequently, these HepG2‐Luc cells were infected with lentiviruses carrying either circ‐shNC or circ‐shRNA constructs. The infection was carried out in the presence of 8–10 µg mL^−1^ polybrene in the culture medium. GFP‐positive cell clones were then selected by serial dilution and further confirmed by real‐time fluorescence quantitative polymerase chain reaction (qRT‐PCR).

### HCC Tissue Collection

HCC tissues and matched adjacent normal tissues were collected from patients undergoing surgery at the First Affiliated Hospital of Xinxiang Medical University and Xinxiang Central Hospital in Xinxiang, China. None of the patients had received adjuvant chemotherapy or radiotherapy prior to surgery. The study was approved by the Ethics Committee of Xinxiang Medical University. All tissue samples were immediately snap‐frozen in liquid nitrogen and stored at −80 °C. Additionally, a tissue microarray containing 24 HCC samples (LWLT‐N‐48LV52) was purchased from Servicebio (Wuhan, China). Fluorescent in situ hybridization (FISH) assays for circZNF79(5) and IF assays for YBX1 and BRCC36 were performed by Servicebio.

### CircRNA Pull‐Down and Mass Spectrometry (MS)

Biotin‐labeled probes targeting the back‐splicing site of circZNF79(5) and a negative control (NC) probe were synthesized by Gene Pharma (Shanghai, China) (sequences listed in Table S4, Supporting Information). MyOne Dynabead Streptavidin C1 beads (Invitrogen, Lot: 91219407, USA) were washed and incubated with cell lysate at 4 °C for 1 h for preclearance. The 3′ biotin‐labeled circZNF79(5) probe or NC probe was then incubated with the beads at room temperature for 3 h to immobilize the probes. Subsequently, the biotinylated beads were incubated with Huh7 cell lysate at 4 °C overnight. After magnetic separation, the beads were washed five times. The beads were then divided into two portions for purification of the pulled‐down proteins or RNA, respectively. The purified proteins were analyzed by MS (Hoogen, China) or western blotting (WB), while the RNA was used for qRT‐PCR analysis.

### Exosome Experiments

Extraction of serum exosomes from 7 pairs HCC and non‐HCC patients was carried out using a Serum and Plasma Exosome Extraction Kit according to the manufacturer's instructions (Umibio, UR52151, China). For cell exosomes, cells were cultured with exosome‐specific serum‐free culture medium (Umibio, UR51102, China) for 48 h, culture medium was collected for exosomes extraction using an Exosome Extraction Kit according to the manufacturer's instructions (Umibio, UR52100, China). Transimission electronic microscopy (TEM) and nanoparticle tracking analysis (NTA) for exosomes were performed by Umibio company (Shanghai, China). For exosomal protein extraction, an equal number of exosomes were homogenized with exosome‐specific lysis buffer (Umibio, UR33101, China). For exosomal RNA extraction, exosomes were pre‐treated with RNase, and an equal number of exosomes were used for RNA extraction.

### RNA Immunoprecipitation (RIP)

For RIP, Protein A/G Agarose Beads (Beyotime, China) were coated with isometric antibodies (IgG or anti‐YBX1 or anti‐AGO2) and incubated with pre‐frozen cell lysates overnight at 4 °C. The RNA‐protein complexes were collected, washed six times, and subjected to proteinase K digestion and RNA extraction using TRIzol. The relative interaction between the protein and RNA was quantified by qRT‐PCR and normalized to the input.

### Co‐Immunoprecipitation (Co‐IP)

Co‐IP was performed using an immunoprecipitation kit (Beyotime, China) according to the manufacturer's instructions. Briefly, 1 mg cell lysate was incubated with the corresponding antibody (anti‐YBX1 or anti‐BRCC36, 1:500, HUABIO, China) or IgG and rotated at 4 °C overnight. The next day, protein A/G Agarose Beads were added and rotated for an additional 2 h. The beads were then collected at 1000 ×g for 1 min and washed at least five times with 1× IP buffer containing a protease inhibitor cocktail (Roche, Switzerland). The beads were eluted with 1× SDS‐PAGE Loading Buffer, heated at 100 °C for 5 min, and subjected to WB analysis.

### Tumor Models

Ten 5‐week‐old male nude mice (Charles River, China) were housed under standard pathogen‐free conditions. For the subcutaneous xenograft model, HepG2‐Luc cells (stably expressing firefly luciferase) transfected with circ‐shRNA or shNC were expanded in culture with puromycin, and injected subcutaneously at 2 × 10^6^ cells in 100 µL serum‐free DMEM mixed with Matrigel (ABW Bio, 0827245, China) (1:1) into the armpit of the right upper limb of each nude mouse. Tumor volume was measured every 6 days. Mice were then sacrificed, tumors were removed and weighed for further studies after 4 weeks.

For the orthotopic xenograft model, intrahepatic injections of 2 × 10^6^ HepG2‐Luc cells in 20 µL serum‐free DMEM mixed with Matrigel (1:1) were performed in the right lobe liver of eight 5‐week‐old male nude mice under sodium pentobarbital anesthesia. Tumor development was monitored weekly using an IVIS system following intraperitoneal injection of 150 mg kg^−1^ D‐Luciferin. And tumors were removed for further studies after 4 weeks.

### Statistical Analysis

Statistical analysis was carried out with SPSS 18.0 software. For statistical evaluation, data were analyzed by two‐tailed Student's t test for comparisons of two samples and one‐way ANOVA for univariate comparisons. Dunnett T3's post hoc test (data with unequal variance) or by Tukey's post hoc test (data with equal variance) was performed when necessary. The GraphPad Prism 8.0 was used for the results demonstration. Image J was used for cell counting and band density measurement. Survival curves were drawn using the Kaplan‐Meier univariate survival analysis and the log‐rank test. Descriptive data were expressed as means±standard errors of means. *P* values < 0.05 were considered statistically significant, ^*^ *P* < 0.05, ^**^ *P* < 0.01, ^***^ *P* < 0.001, ^****^ *P* < 0.0001, and *P* > 0.05 not significant (*ns*).

### Ethics Approval and Consent to Participate

This study was approved by the Ethics Committee of Xinxiang Medical University (Henan, China) under approval numbers XYLL‐20220080 (animal experimental procedures) and XYLL‐20230040 (human HCC study). All experiments involving human subjects were conducted in accordance with the Declaration of Helsinki and relevant institutional guidelines.

## Conflict of Interest

The authors declare no competing interests.

## Author's Contribution

X.G. and L.X. contributed equally to this work. X.Q.G., L.L.X., Y.K.L., and W.B.L. performed experiments and analyzed data. A.D.J. and K.Y. collected the HCC clinical tissues. L.W., Z.L.F., Y.P.S. and J.H.Z. provided technical advice about experimental design and joined the discussion. T.H. and K. W. C. provided some of the plasmids used in this experiment. Z.Q., W.Y.W., Z.L., and W.J.S. participated in the analysis of experimental data. T.Z.L. and S.P.M. made contributions to the revision of the manuscript. X.Q.G., L.L.X., C.S.X., W.S.G., and W.J.R. initiated the study, designed experiments, wrote the manuscript, and prepared the final scheme presentation.

## Supporting information



Supporting Information

Supplemental Video 1

## Data Availability

The data that support the findings of this study are available in the supplementary material of this article.
