# Supplementary material for: Dysregulation of CircZNF79(5) Modulates YBX1 Stability and Selective Autophagy to Drive Hepatocellular Carcinoma Progression
Source: Adv Sci (Weinh). 2025 Nov 12;13(6):e10310. doi: 10.1002/advs.202510310 (PMC12866679; doi:10.1002/advs.202510310)
Supplement: Supplementary file 1 — Supporting Information [file ADVS-13-e10310-s001.docx]

**Supplementary Materials**

**Dysregulation of circZNF79(5) Modulates YBX1 Stability and Selective Autophagy to Drive Hepatocellular Carcinoma Progression**

Xueqiang Guo^1, 4#^, Lingling Xi^1, 2#^, Yukun Liu^1^, Wenbao Lv^1^, Tianzi Li^1^, Andong Ju^3^, Zhenlin Fan^1, 4^, Yaping Shen^1^, Zhuang Qian^4^, Weiyun Wang^1^, Zhuo Liang^4^, Wenjuan Song^1^, Kaiwen Chang^1^, Shuangping Ma^1^, Junhe Zhang^1^, Tao Han^1^, Kun You^5^, Cunshuan Xu^6^, Lei Wang^4*^, Weisheng Guo^4, 7*^, Wenjie Ren^1, 4*^

Correspondence to: Wenjie Ren (wjren1966@163.com), Weisheng Guo (guo_wei_sheng@gzhmu.edu.cn), Lei Wang (lwang0522@163.com)

^#^ These authors contributed equally to this work.

**This file includes:**

Supplementary Materials and Methods

Supplementary Tables S1 to S4

Supplementary Figures S1 to S22

**Materials and Methods**

***Cell culture and treatments***

Human HEK293T cell, HCC cell lines (Huh7, HepG2, Bel-7402 and MHCC-97H) from ATCC, and the human fetal hepatocyte line (HL7702) obtained from Henan normal University were cultured in the DMEM-High glucose medium (Gibco, USA) containing 10% serum (Gibco, USA) and 1% penicillin/streptomycin (Gibco, USA) in a humidified incubator (37 °C, 5% CO_2_, 95% humidity). Cells were routinely tested for mycoplasma.

The follow reagents were used for cells treatments: Dimethyl sulfoxide (DMSO) (SigmaAldrich, Cat# D8418), 200 μg/mL Cycloheximide (CHX, AbMole, M4879), 20 μM MG132 (MedChemExpress, HY-13259), 20 μM Chloroquine (CHQ, MedChemExpress, HY-17589A), 50 μM 3-Methyladenine (3-MA, MedChemExpress, HY-19312), 20 nM Rapamycin (RAPA, MedChemExpress, AY-22989), All drug experiments were started at 12 h after cells were seeded or transfected. Cells were exposed to fresh MEM complete medium containing drugs or control DMSO for the indicated time and then washed with PBS for 3 times.

***Plasmids and transfection***

Two specific siRNAs of circZNF79(5) targeting the junction region sequence, YBX1, p62 and BRCC36 were synthesized by GenePharma (Shanghai, China), and related sequences are recorded in Table S4 (Supplementary Table S4). To establish the stable circZNF79(5)-knockdown HCC cell lines, the short hairpin circZNF79(5)-shRNA sequence (based on the same sequence as circZNF79(5)-si1) was cloned into pGLV3/H1/GFP-Puro or LV2N(U6/Puro) vector. The LV5(EF-1a/GFP&Puro) vector (GenePharma, Shanghai, China) was used to construct the circZNF79 overexpression plasmid. Coding sequences of human YBX1-3×Flag (NCBI Ref Seq: NM_004559.5), YBX1-GFP, YBX1-Delet (1-60)-3×Flag-GFP, YBX1-Delet (61-125)-3×Flag-GFP, and YBX1-Delet (126-324)-3×Flag-GFP were cloned into pCDNA3.1 by Kelei biological Technology (Shanghai, China), LC3-GFP-mcherry and LC3-mcherry constructs were provided by Prof. Qiwen Wang from College of Life Sciences, Henan Normal University, China. The HA-Ub-WT, -K48, and-K63 constructs were provided by Prof. Tao Han from Institutes of Health Central Plain, Xinxiang Medical University, China. Transfections of plasmids or siRNA was performed using Lipofectamine® 3000 (Invitrogen, USA) according to the manufacturer’s protocol.

***CCK8 assay and colony formation assay***

For CCK8 assay, HCC cells were seeded into 96-well plates at at a density of 3000/well, and the viability of HCC cells was determined by Cell Counting Kit 8 (Biosharp, China) and measured at OD_450 nm_ with the Varioskan Flash system (Thermo, USA). For colony formation assay, 6-cm dishes were seeded with 1 × 10^4^ viable HCC cells (Huh7 and HepG2) infected with shcircZNF79(5) or shNC virus and allowed to grow for 10 days. The colonies obtained were washed gently with PBS and fixed in 4% paraformaldehyde for 20 minutes at room temperature and then washed with PBS followed by staining with 0.1% (w/v) crystal violet. The colonies with >50 cells under the microscope were counted. Three different independent experiments were performed.

***EdU incorporation assay***

An EdU incorporation assay was carried out using a Cell-Light EdU imaging detection kit according to the manufacturer's instructions (C10310, RiboBio, Guangzhou, China). Briefly, the cells were seeded and treated with the methods as above and 48 h later, cells were initially incubated with 50 μmol/L of EdU for 2h at 37°C. After being fixed with 4% paraformaldehyde for 30 min, anti-EdU working solution was added at room temperature in the dark for 30 min and incubated with 5 μg/mL of Hoechst 33,342 to stain the cell nuclei for 10 min. Images were captured by a fluorescent microscope (Zeiss, Germany). Three different independent experiments were performed.

***Wound healing assays***

Cells were seeded at a density of 2 × 10^6^ /well in a 6-well plate, and incubated until 100% confluence. The layer of cells was scratched with a 10 µL pipette tip (Sigma), and washed three times with PBS and incubated with fresh serum-free DMEM medium. Images were taken at 0, 24, and 48h after wounding with microscope (Zeiss, Germany) and cell motility was assessed through the distance between the wound edges by statistical analysis.

***Migration and invasion assay***

Cell migration and invasion assays were performed using Transwell units with 6.5 µm-pore polycarbonate filters (Corning Incorporated, Corning, NY, USA). For the migration assay, cells cultured in 150 μL DMEM were seeded in the upper chamber, while 500 μL medium with 10% FBS was put in the lower chamber. Cells were cultured for 24 h, the cells in the upper chamber were fixed and stained with crystal violet. Cells in the upper chamber were removed with a cotton swab and the number of cells that migrated across the membrane was counted in five randomly selected microscopic fields in at a minimum of three independent experiments. For the invasion assay, a similar protocol was performed using Matrigel-coated (BD Biosciences) instead of Transwell chambers. The cells that suspended into the well bottom were quantified after 48 h incubation at 37˚C. Cells in the upper chamber were subjected to microscopic inspection.

***Flow cytometry for cell cycle analysis***

The cell cycle was evaluated using a flow cytometry (FCM). Briefly, the cells were seeded in a 6 well plate at a density of 2×10^5^ cells/well and harvested at 48 h. After two washes in cold PBS, the samples were fixed in 70% ethanol for 12 h on ice, which was followed by staining with 1 mL of a PBS solution with 20 μg propidium iodide (PI, Sigma, USA) and 50 μg of RNase A (Sigma, USA) for 30 min at 37°C. The samples were then analyzed for DNA content by BD Accuri C6 Plus (USA). Three independent experiments were performed for each group.

***Flow cytometry for cell apoptosis***

Apoptosis rate was measured as the following procedures. At 48 h after transfection, cells were harvested using flow cytometry tube and centrifuged for 5 min at 1000 r/min at 4°C, followed by the removal of supernatant. Then the cells were washed in cold PBS for three times and centrifuged, with supernatant aspirated. In accordance with the in struction of Annexin V-FITC apoptosis detection kit (Beyotime, C1062, China), 100 μL binding buffer and 3.5 μL Annexin-V-FITC and 3.5 μL PI were added into each tube and well-mixed by shaking, followed by 30 min incubation in the dark. Finally, 400 μL 1×binding buffer were added to the cells, which were analyzed by BD Accuri C6 Plus (USA).

***qRT-PCRs, RNase R treatment and Actinomycin D (ActD) assays***

Total RNA was extracted with Trizol reagent (Biosharp, China), and cDNA was obtained using Reverse Transcription System Kit (Vazyme, R312, China). The primers for the genes were designed by Primer Express 5.0 software (Supplementary Table 2). qRT-PCR was performed with 2× Universal SYBR qPCR Master mix (Vazyme, Q712, China) using the Step One Plus Real-Time PCR system (Cobas z 480, Roche). β-actin was employed as an internal reference and GAPDH was used as an internal reference for mRNAs localized in cytoplasm. Each sample was performed in triplicates. The relative expression of target genes was calculated with the 2^-ΔΔ^Ct method. For RNase R treatment, 1 unit of RNase R (Geneseed, R0301, China) was added to digest 1 μg of RNA for 15 min at 37 °C. The ActD assay was used to verify the stability of circZNF79(5) and ZNF79 mRNAs. Huh7 were incubated with 10 μg/mL ActD (AbMole, M4881) for 0, 4, 8 and 12 h, respectively. qRT-PCR was used to determine the remaining relative circRNA and linear ZNF79 mRNA.

***Western blot analysis***

After treatment, cells were homogenized in RIPA lysis buffer (Beyotime Biotechnology, #P0013B, China) containing protease and phosphatase Inhibitors Cocktail (Roche) and quantified by BCA protein assay kit (Beyotime, #P0009, China). Equal amounts of proteins were subjected to SDS-PAGE and transferred to PVDF membranes (Millipore, USA) at 100 mA (overnight, 4℃). The membranes were blocked in 5% (w/v) skimmed milk in TBS containing 0.1% (v/v) Tween-20 (TBST) for 1h and then treated with the primary antibodies overnight at 4℃. After three washes in cold PBS, immunoreactivity was revealed using a suitable horseradish peroxidase (HRP) labeled secondary antibody and signals were detected using an ECL kit (RM00020P, ABclonal, China) by a ChemiScope6100 Imaging System (CLINX, China). The band density was measured using Image Quant TL software. GAPDH was served as an internal reference. List of antibodies used for western blot was shown in Supplementary Table 3.

***Cytosolic/Nuclear Fractionation***

Cells were washed and harvested in cold PBS. The cytoplasmic and nuclear fractions were then separated by using a Nuclear and Cytoplasmic Protein Extraction Kit (Beyotime, Shanghai, China, #P0028) according to the manufacturer’s instructions. Protein concentrations were determined by using BCA protein assay kit, and then subjected to immunoblotting and qPCR, with GAPDH as cytoplasmic and U6 as nuclear references.

***Fluorescent in situ hybridization (FISH)***

The cy3-labeled ﬂuorescent probes targeting circZNF79(5) was synthesized by GenePharma (Shanghai, China), and their sequences are listed in Table S4. Brieﬂy, Huh7 and HepG2 cells (5 × 10^3^) were seeded in confocal dishes, ﬁxed with 4% paraformaldehyde, and permeabilized with 0.4% Triton X-100. Then the cells were hybridized with labeled probes in the reaction buffer over night at 37°C. Subsequently, the cell nucleus was stained with DAPI and photographed by Stimulated Emission Depletion microscopy (Leica TCS SP8 STED).

***Immunofluorescent staining (IF)***

After indicated treatments in the figure legends, cells were fixed with 4% paraformaldehyde for 15 min at room temperature, permeabilized with 0.4% Triton X-100 for 5 min, and blocked with 5% BSA in PBS for 1 h. Cells were then incubated with anti-YBX1 (1:500, Abcam, ab76149) and anti-BRCC36 (1:500, HUABIO, PSH05) overnight at 4 °C followed by three times wash with PBS, and then incubated with Alexa Fluor 594 goat anti-Rabbit IgG secondary antibody (1:1000, Invitrogen, A11012), Alexa Fluor Plus 647 goat anti-Rabbit IgG secondary antibody (1:1000, Invitrogen, A21244) for 3 h at 4°C followed by another three washed with PBS. Cells were then stained with DAPI to visualize the nuclei. Images were taken by microscopy (Leica TCS SP8 STED).

***Hematoxylin-eosin (H&E) and immunohistochemical (IHC) staining***

Tissues were fixed in 10% (v/v) formaldehyde in PBS, embedded in paraffin, and cut into 5 μm sections and used for H&E staining and IHC staining with specific primary antibodies against YBX1 and Ki67. To enhance antigen exposure, the slides were treated with 1 × EDTA at 98° C for 10 min for antigen retrieval. The slides were incubated with endogenous peroxidase blocking solution and then were incubated with the primary antibody at 4℃ overnight. After rinsing with Tris-buffered saline, the slides were incubated for 45 min with biotin-conjugated secondary antibody, washed, and then incubated with enzyme conjugate horseradish peroxidase (HRP)-streptavidin. Freshly prepared DAB (Zymed, South San Francisco, CA) was used as a substrate to detect HRP. Finally, slides were counter-stained with hematoxylin and mounted with aqueous mounting media and and images were captured.

***Label-free LC-MS/MS***

The Huh7-circ-shNC and circ-shRNA cell samples were sent to Hoogen biotech (Shanghai, China) for proteomic sequencing, and the Top5 signaling pathways with the lowest p-value were screened by bioinformatics methods such as Gene Ontology (GO) and Kyoto Encyclopedia of Genes and Genomes (KEGG) and GSEA database, and the results of high-throughput sequencing were verified by WB.

**Supplementary Tables S1 to S4**

**Table S1.** Association between clinical features and circZNF79 expression of hepatocellular carcinoma patients.

| **Clinical features** | **circZNF79 expression** | | ***P* value** |
| --- | --- | --- | --- |
|  | **Low (< median)** | **High (> median)** |  |
| **Number** | 11 | 13 |  |
| **Sex** |  |  | 0.3879 |
| Male | 7 | 11 |  |
| Female | 4 | 2 |  |
| **Age(years)** |  |  | 0.9109 |
| <50 | 6 | 7 |  |
| >50 | 5 | 6 |  |
| **Tumor size(cm)** |  |  | 0.0341* |
| <6 | 5 | 5 |  |
| >6 | 6 | 8 |  |
| **TNM stage** |  |  | 0.0284* |
| I+II | 7 | 3 |  |
| III+IV | 4 | 10 |  |
| **Intrahepatic metastasis** | |  | 0.0432* |
| Yes | 7 | 10 |  |
| No | 4 | 3 |  |

**Table S2. Sequences of primers in this study**

| **Gene name** | **Primer Sequence (5' to 3')** | **Product size (bp)** |
| --- | --- | --- |
| *β‑actin* | F: ACATCCGTAAAGACCTCTATGCCAACA | 109 |
|  | R: GTGCTAGGAGCCAGGGCAGTAATCT |  |
| *GAPDH* | F: CACGGCAAGTTCAACGGCACAGTCA | 152 |
|  | R: GTGAAGACGCCAGTAGACTCCACGAC |  |
| *U6* | F: CTCGCTTCGGCAGCACA |  |
|  | R: AACGCTTCACGAATTTGCGT |  |
| circZNF79- convergent | F: ATGTGTAGAGATGCCCCC | 168 |
|  | R: GGTTTCAGGATTTCCGAGTT |  |
| circZNF79- divergent | F: GCTCTGCCTTCGTTAGACATCA | 216 |
|  | R: TCTTCTCAAGGTCACTGGTCCC |  |
| *ZNF79* | F: TTCAAGGAGGGGATACCAGG | 338 |
|  | R: TGTGTCTCACATTTGCGAGG |  |
| *YBX1* | F: TGAGGCAGAATATGTATCGGGG | 203 |
|  | R: GCCATCTTGTGGTTTAGGGTTT |  |

**Table S3. Antibodies used in this study**

| **Antibody** | **Company (Art.No.)** | **Apply (dilution)** | **MW (kDa)** |
| --- | --- | --- | --- |
| GAPDH | Affinity (AF7021) | WB (1:5000) | 36 |
| YBX1 | Abcam (ab76149)  HUABIO (ST0432) | IF (1:500)  IHC (1:500)  WB (1:2000)  IP (1:500) | 50 |
| LC3Ⅰ/Ⅱ | Abways technology (CY5992) | WB (1:1000) | 14/16 |
| p62 | ABclonal (WH136110) | WB (1:1000) | 62 |
| Flag-tag | ABclonal (WH135788) | WB (1:5000) |  |
| BRCC36 | HUABIO (PSH05) | IF (1:500)  WB (1:2000) | 36 |
| HA-tag | HUABIO (PSH01-92) | WB (1:2000) |  |
| Ki67 | HUABIO (HA721115) | IHC (1:5000) |  |
| HIF-1a | Affinity (BF8002) | WB (1:2000) | 90/120 |
| OPTN | OPTN | WB (1:2000) | 66 |
| NDP52 | HUABIO (HA721356) | WB (1:2000) | 52 |
| Ubiquitin specific K48 | Abcam (ab140601) | WB (1:1000) |  |
| Ubiquitin specific K63 | Abcam (ab179434) | WB (1:1000) |  |
| AMPK | CST (5831T) | WB (1:2000) | 62 |
| p-AMPK (Thr172) | CST (2535T) | WB (1:1000) | 62 |
| m-TOR | Abways technology (CY5306) | WB (1:2000) | 289 |
| p-m-TOR(S2448) | Proteintech (NO: 6778) | WB (1:1000) | 289 |
| TSG101 | Aladdin (ab132811) | WB (1:2000) | 44 |
| CD9 | Aladdin (ab095484) | WB (1:2000) | 25 |
| CD63 | Aladdin (ab095139) | WB (1:2000) | 26/45 |
| Calnexin | SciBen(61461) | WB (1:2000) | 90 |
| PCNA | Affinity (AF0239) | WB (1:5000) | 36 |
| CCND1 | Wanleibio(WL01435a) | WB (1:2000) | 36 |
| AGO2 | HUABIO (ET1702-39) | WB (1:2500)  IP (1:500) | 97 |
| Goat Anti-Mouse IgG | ProteinBiotechnologies(Cat#PMS301) | WB (1:3000) |  |
| Goat Anti- Rabbit IgG | ProteinBiotechnologies(Cat#PMS302) | IHC (1:500)  WB (1:3000) |  |
| Alexa Fluor 594 goat anti-Rabbit IgG | Invitrogen (A11012) | IF (1:1000) |  |
| Alexa Fluor 647 goat anti-Rabbit IgG | Invitrogen (A21244) | IF (1:1000) |  |

**Table S4. Sequences of specific siRNA targets in this study**

| **Gene name** | **Primer Sequence (5' to 3')** |
| --- | --- |
| circZNF79-si1 | Sence: CUCAGGACAGGCUGGAAGATT |
|  | Anti sence: UCUUCCAGCCUGUCCUGAGTT |
| circZNF79-si2 | Sence: GACUCAGGACAGGCUGGAATT |
|  | Anti sence: UUCCAGCCUGUCCUGAGUCTT |
| BRCC36-si1 | Sence: GGACCGAGUAGAAAUUUCUTT |
|  | Anti sence: AGAAAUUUCUACUCGGUCCTT |
| BRCC36-si2 | Sence: GGCCUUCACAUGUUGAUGUTT |
|  | Anti sence: ACAUCAACAUGUGAAGGCCTT |
| YBX1-si1 | Sense: GGAGGCAGCAAAUGUUACATT |
|  | Antisense:UGUAACAUUUGCUGCCUCCTT |
| YBX1-si2 | Sense:GGACGGCAAUGAAGAAGAUTT |
|  | Antisense:AUCUUCUUCAUUGCCGUCCTT |
| p62-si1 | Sense:GAUGACAUCUUCCGAAUCUTT |
|  | Antisense:AGAUUCGGAAGAUGUCAUCTT |
| p62-si2 | Sense:CAUCCAGUAUUCAAAGCAUTT |
|  | Antisense:AUGCUUUGAAUACUGGAUGTT |
| siNC | Sense: UUCUCCGAACGUGUCACGUTT |
|  | Antisense: ACGUGACACGUUCGGAGAATT |
| SNC | UUGUACUACACAAAAGUACUG |
| SAO | UGUUUGGUGAGGUUGGCGUU-3' cy3 |
| circZNF79(5)-probe | AATCTTCCAGCCTGTCCTGAGTCGG-3' Biotin |
|  | AATCTTCCAGCCTGTCCTGAGTCGG-3' cy3 |

**Supplementary Figures and Legends**

**
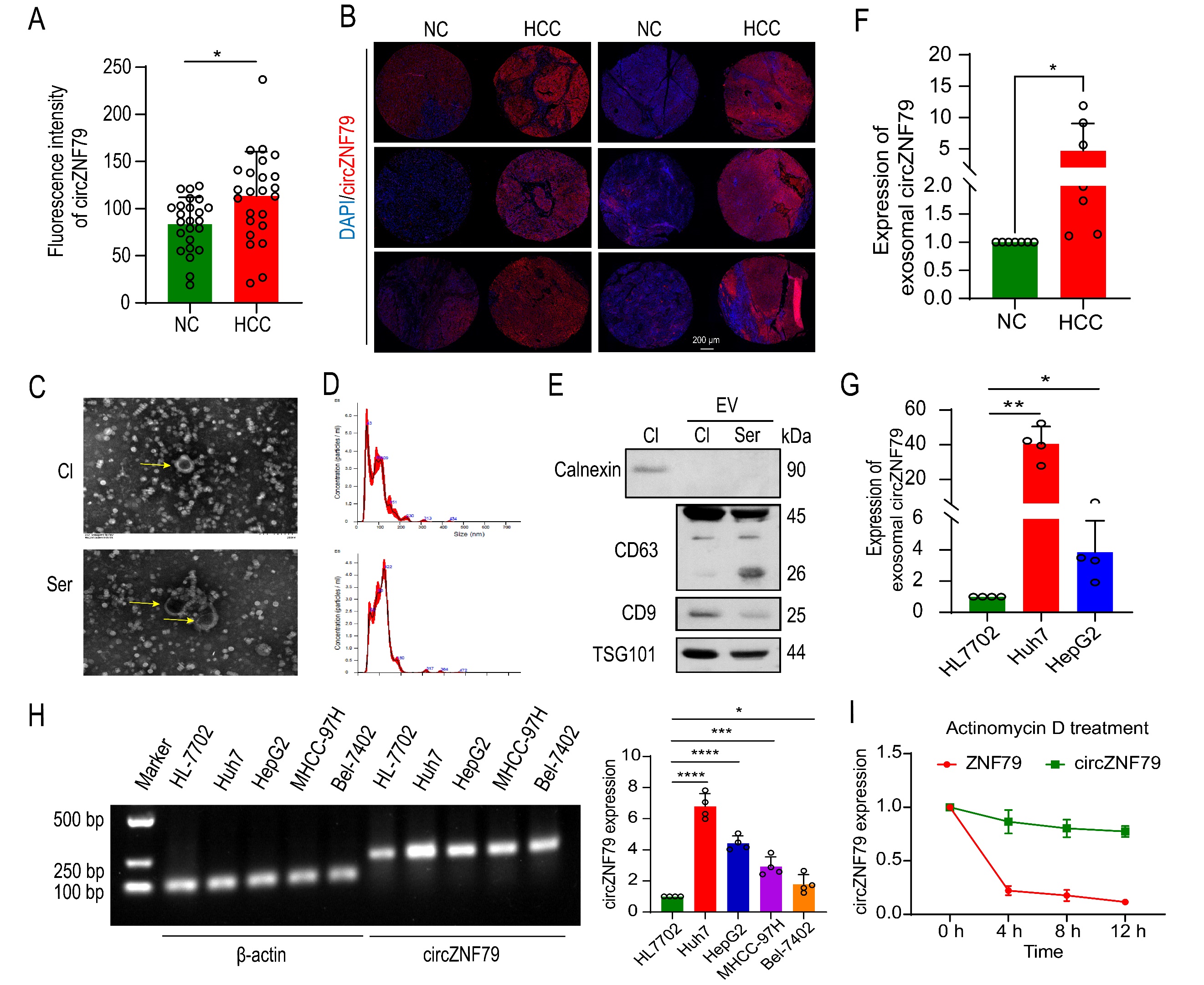
**

**Figure S1.** **circZNF79(5) is overexpressed in HCC tissues, cells, and exosomes from serum and culture media of cells. A&B**, FISH detected and statistical analyzed the fluorescence intensities of circZNF79(5) expression in 24 HCC samples tissue array (A), and showed 6 of them randomly (B). Scale bar = 200 μm. **C-E**, Exosome were identified by transmission electron microscopy (C), particle size analysis (D) and biomarkers, including TSG101, CD9, CD63 and Calnexin (E), Cl means cell and Ser means serum. **F&G**, Exosomal circZNF79(5) expressions in the blood serum of 7 pairs HCC and non-HCC patients (F) and culture media of Huh7 and HepG2 cells (G) detected by qRT-PCR, *n* = 4. **H**, Expression and statistical analysis of circZNF79(5) in normal cell line (HL7702) and HCC cell lines (Huh7, HepG2, Bel-7402 and MHCC-97H) detected by qRT-PCR, *n* = 3. **I**, The half-life of circZNF79(5) and linear ZNF79 mRNA after Actinomycin D (ActD) treatment, *n* = 4. Statistical analysis was performed using a two-tailed t-test, **P* < 0.05, ***P* < 0.01, ****P* < 0.001, *****P* < 0.0001. Data are presented as mean ± S.E.M.

**
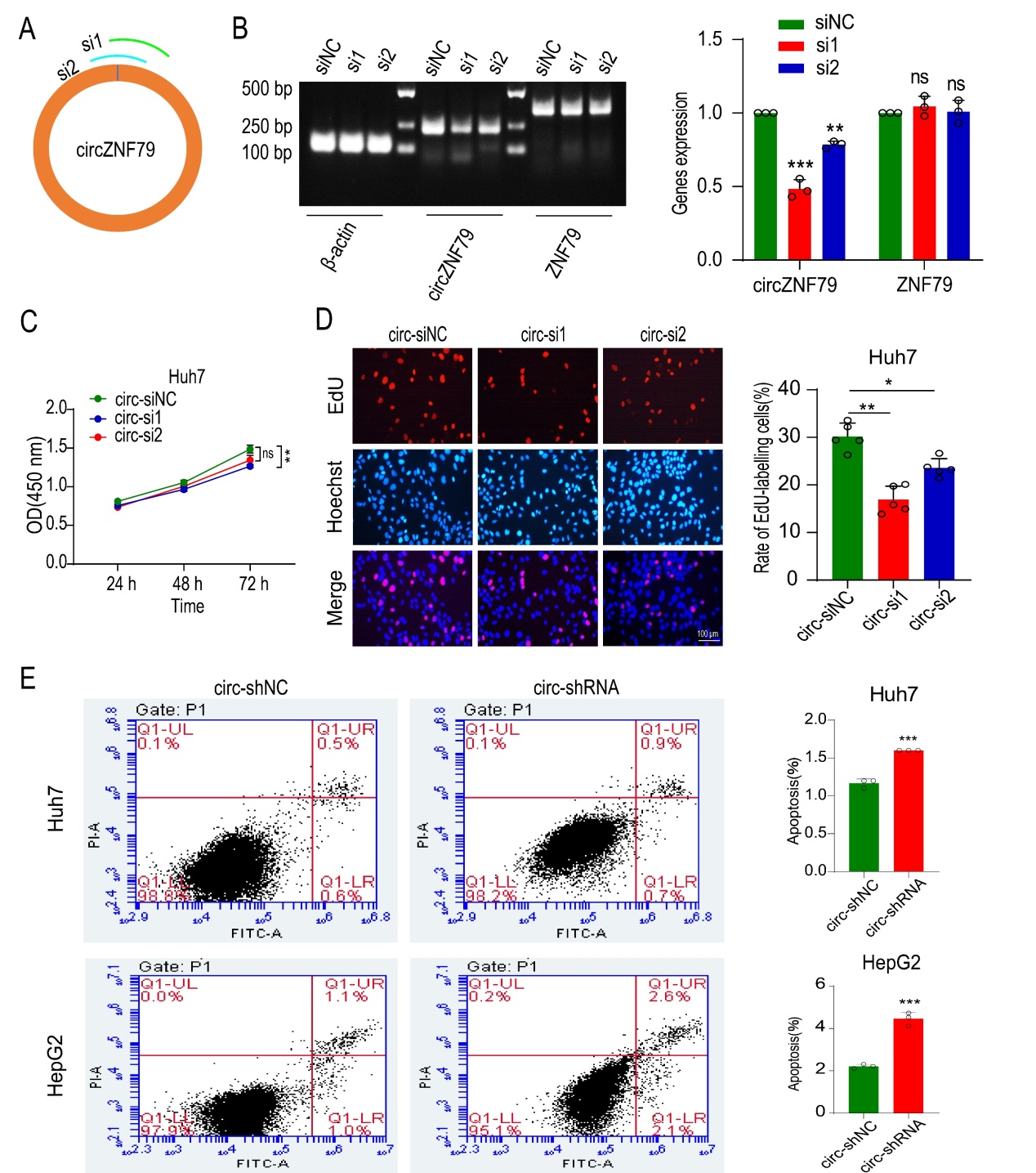
**

**Figure S2.** **Knockdown circZNF79(5) inhibits HCC cell proliferation, migration, but promotes apoptosis *in vitro*. A**, Schematic representation of where the siRNAs targeting circZNF79(5) were designed. **B**, qPCR analysis of circZNF79(5) expression after transfecting Huh7 cells with siNC, si1 and si2, n=3. **C**, CCK8 assay of Huh7 cells transfected with siNC, si1 or si2, *n*=5. **D**, EdU assay of Huh7 cells transfected with siNC, si1 or si2, *n*=5. Scale bar = 100 μm. E, Flow cytometry and statistical analysis for cell apoptosis of Huh7 and HepG2 cells transfected with circ-shNC or circ-shRNA, n=3. Three independent experiments with three technical repetitions were performed. Statistical analysis was performed using a two-tailed t-test, **P* < 0.05, ***P* < 0.01, ****P* < 0.001, and *P* > 0.05 not significant (*ns*). Data are presented as mean ± S.E.M.


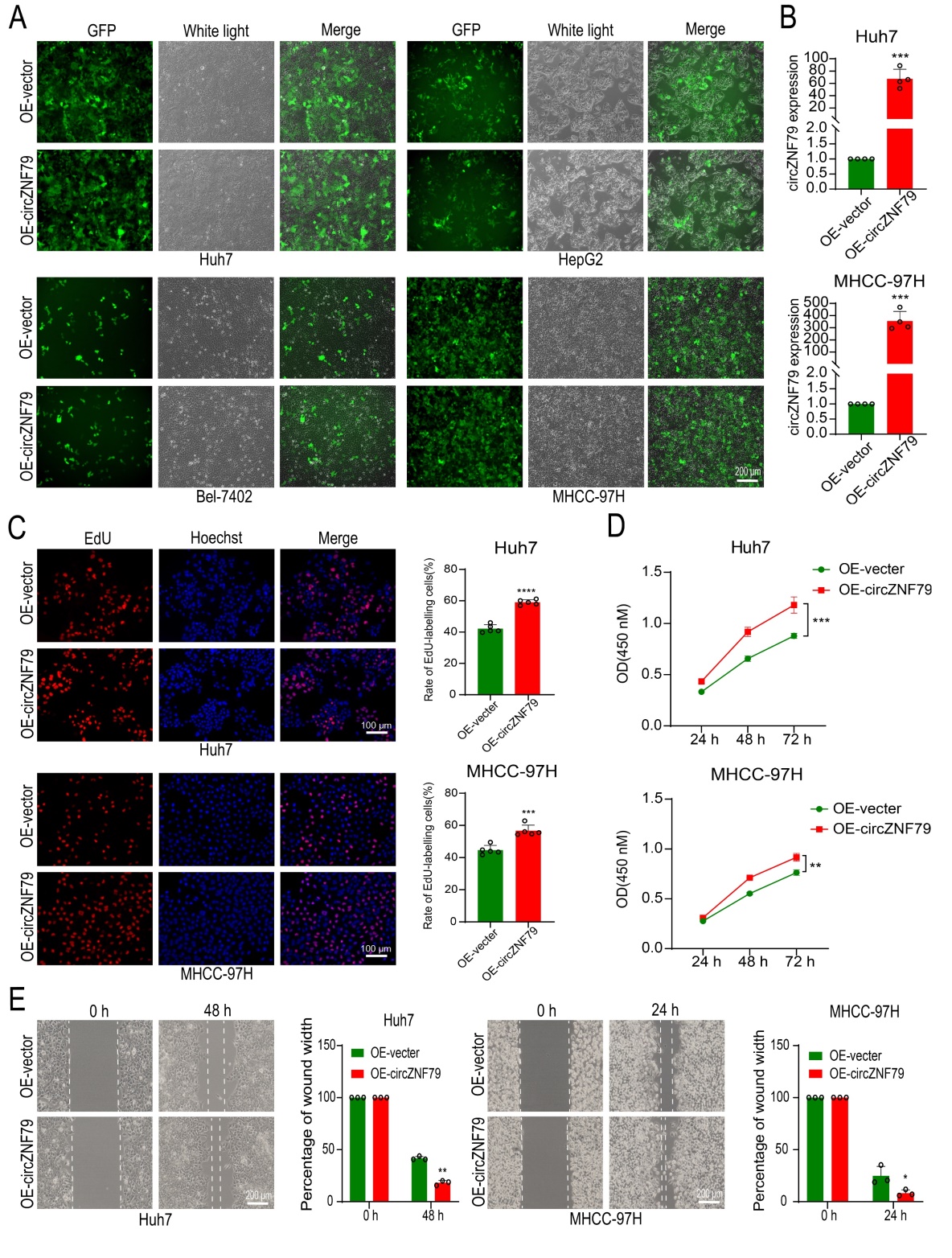


**Figure S3.** **Overexpression circZNF79(5) promotes HCC cell proliferation, migration in vitro. A**, The circZNF79(5) overexpression effect by LV5(EF-1a/GFP&Puro) vector transfection in HCC cell lines (Huh7, HepG2, Bel-7402 and MHCC-97H). **B-E**, circZNF79(5) expression detected by qRT-PCR (B, n = 4), EdU (C, n = 5), CCK8 (D, n = 3) and wound healing assays (E, n = 3) in highly infectious Huh7 and MHCC-97H cells transfected with OE-vector or OE-circZNF79(5). Three independent experiments with three technical repetitions were performed. Statistical analysis was performed using a two-tailed t-test, **P* < 0.05, ***P* < 0.01, ****P* < 0.001, and *****P* < 0.0001. Data are presented as mean ± S.E.M.


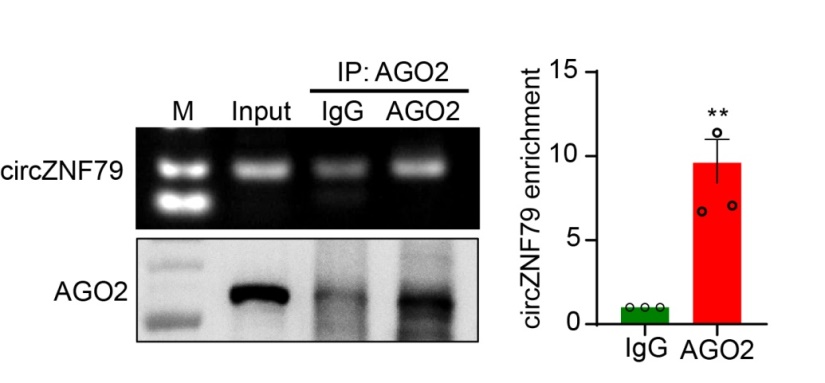


**Figure S4. circZNF79(5) can bind to AGO2.** RIP assays showed that circZNF79(5) can bind to AGO2. M means marker. Three independent experiments with three technical replicates were performed. Statistical analysis was conducted using a two-tailed *t*-test. ***P* < 0.01, n = 3. Data are presented as mean ± S.E.M.


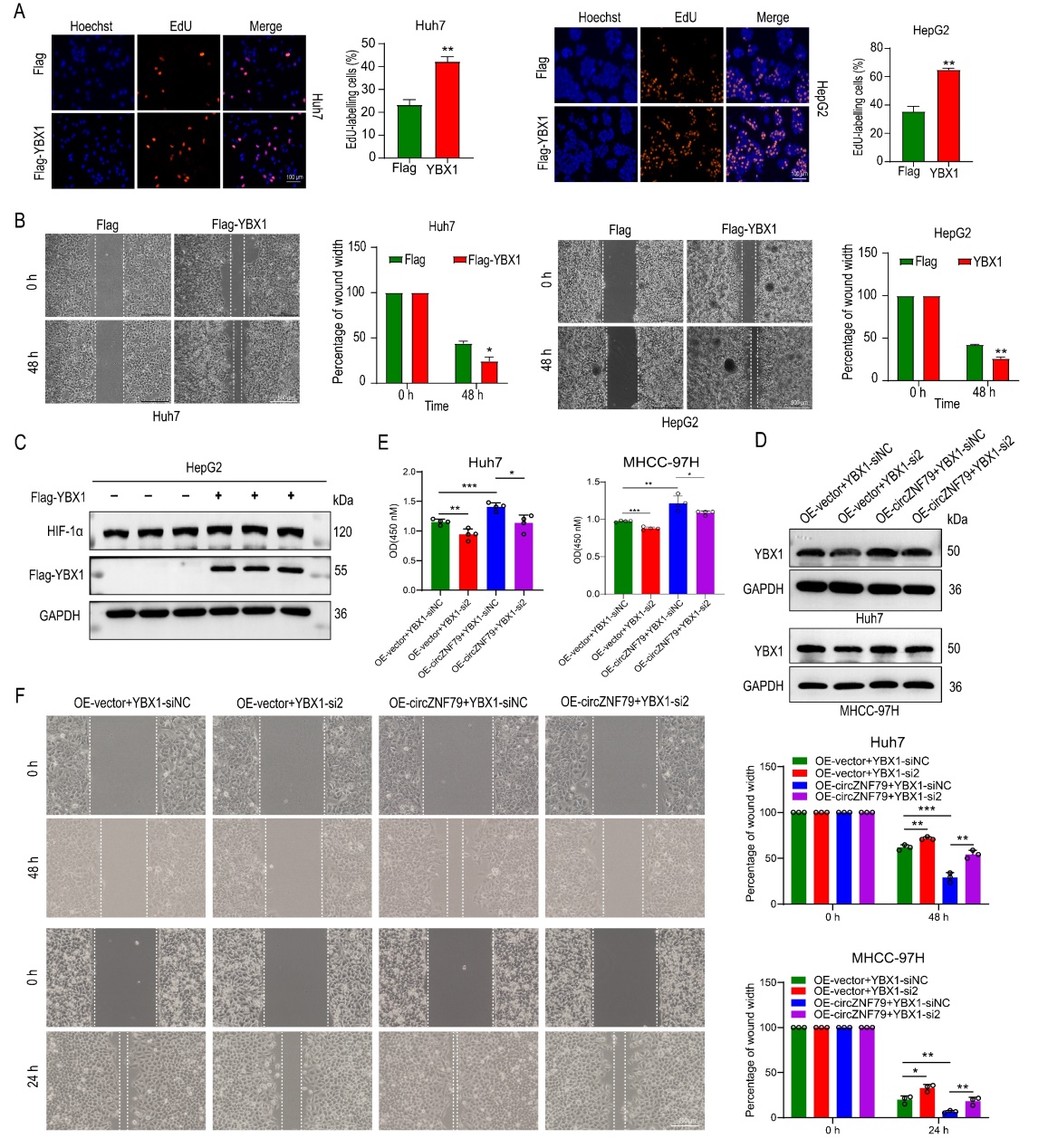


**Figure S5.** **YBX1 mediates circZNF79(5) oncogenic function on HCC cell proliferation and migration. A**, The EdU assays of HCC cells following with Flag -YBX1 or Flag plasmids transfection. *n*=3, scale bar = 100 µm. **B**, Wound healing assays of HCC cells transfected with Flag-YBX1 or Flag plasmids, *n*=3, scale bar = 500 µm. **C**, WB analysis of YBX1, HIF-1α and GAPDH in HepG2 cells transfected with YBX1-Falg or Flag-vector, *n*=3. **D**, WB analysis of YBX1 and GAPDH in Huh7 and MHCC-97H cells transfected with YBX1-siNC or -si2, and OE-vector or OE-circZNF79(5), n=3. **E&F**, Functional restoration experiments: YBX1 knockdown reversed circZNF79(5) overexpression-induced proliferative and migratory promotion in Huh7 and MHCC-97H cells by CCK8 assay at 72 h (E, n = 4) wound healing assays (F, n = 3, Scale bar = 200 µm). Three independent experiments with three technical repetitions were performed. Statistical analysis was performed using a two-tailed t-test, **P* < 0.05, ***P* < 0.01, and ****P* < 0.001. Data are presented as mean ± S.E.M.


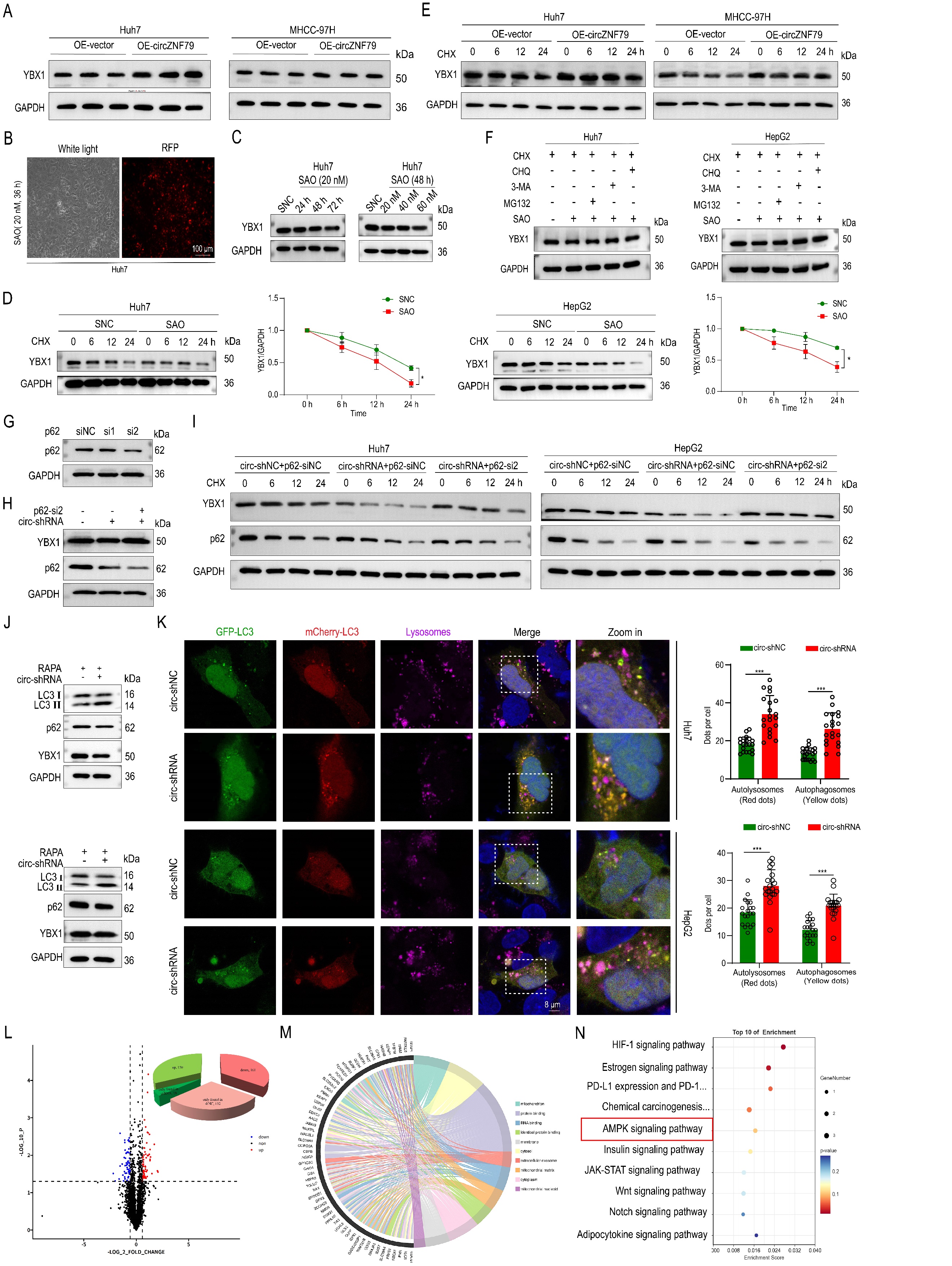


**Figure S6.** **CircZNF79(5) interacts with YBX1 to inhibit its autophagic degradation *via* AMPK signaling pathway.** **A**, WB analysis of YBX1 and GAPDH in Huh7 and MHCC-97H cells transfected with OE-vector or OE-circZNF79(5), n=3. **B**, Fluorescence images showed that SAO was successfully transferred into Huh7 cells. Scale bar = 100 μm. **C**, WB assay showed that disrupting the binding of YBX1 and circZNF79(5) using SAO decreased the protein levels of YBX1, which showed time and concentration dependence. **D**, CHX chase assays showed that YBX1 protein stability was decreased in HCC cells transfected with SAO. The protein levels of YBX1/GAPDH were quantified by ImageJ software. **E**, CHX chase assays showed that YBX1 protein stability was increased in HCC cells transfected with OE-circZNF79(5). **F**, WB analysis of YBX1 stabilization in HCC cells transfected with SAO or SNC, followed by DMSO, MG132 (20 μM), 3-MA (50 μM) or CHQ (20 μM) treatment for 6h. **G**, WB analysis of p62 and GAPDH in Huh7 cells transfected with p62-siNC, -si1 or –si2, n=3. **H**, WB analysis of YBX1, p62 and GAPDH in Huh7 cells transfected with p62-siNC or –si2 and circ-shNC or -shRNA, n=3. **I**, WB analysis of YBX1, p62 and GAPDH in HCC cells transfected with p62-siNC or –si2 and circ-shNC or –shRNA, and treated with CHX (200 µg/mL) at 0, 6, 12, and 24 hours, n=3. **J**, WB analysis of LC3 and p62 in shNC and shRNA cells treated with RAPA (20 nM) for 6 h. **K**, Fluorescence microscopy shows the formation of GFP-mCherry-LC3 puncta in HCC circ-shNC or -shRNA cells treated with RAPA (20 nM) for 6 h. *n*=20. **L-N**, Bioinformatics analysis, including Volcano (**L**), up/down-regulated genes number (top right corner of L), GO (M) and KEGG (N) analysis of differential expression protein in HepG2 cells transfected with circ-shNC or -shRNA. Three independent experiments with three technical repetitions were performed. Statistical analysis was performed using a two-tailed t-test, **P* < 0.05, and ****P* <0.0001. Data are presented as mean ± S.E.M.


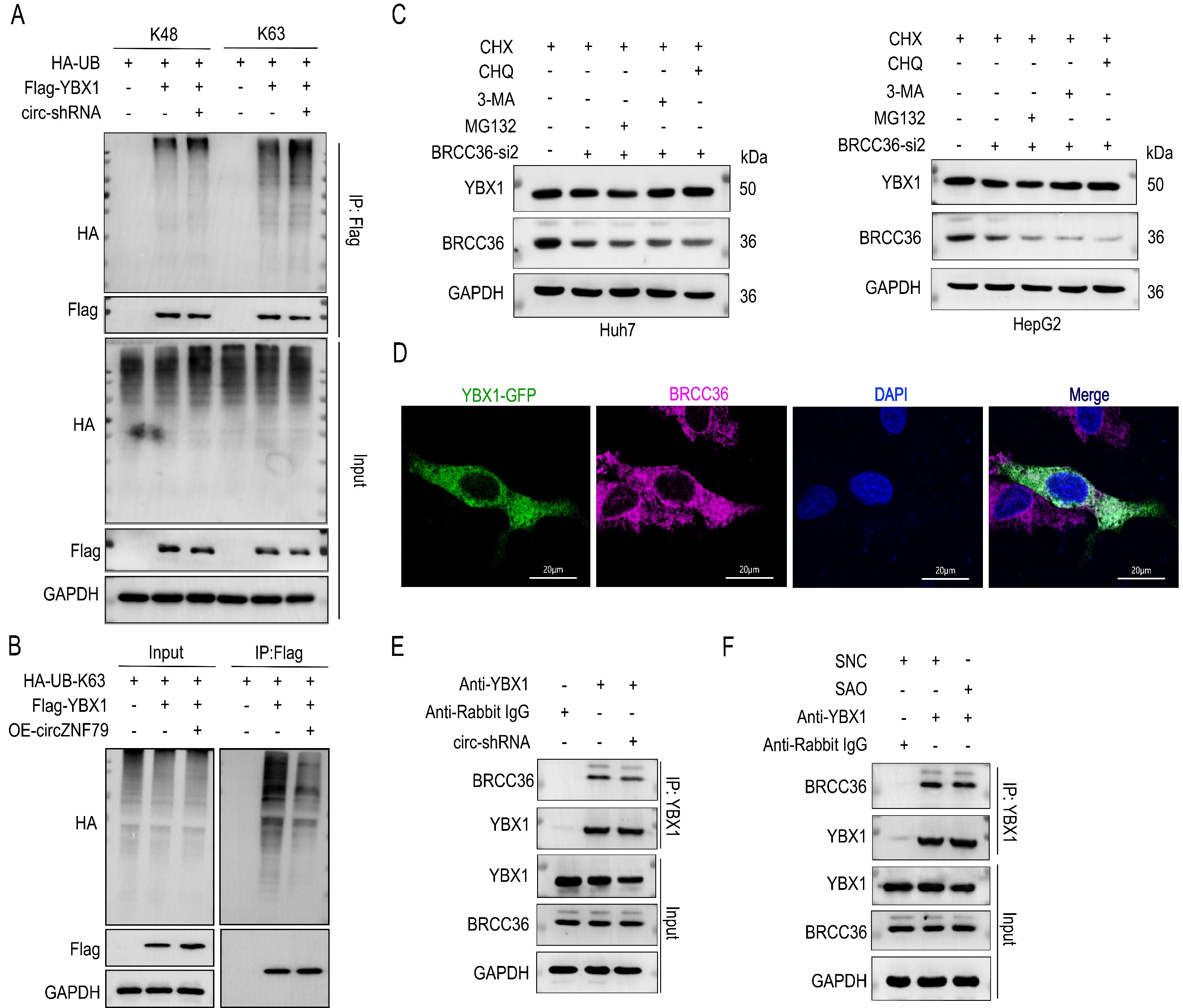


**Figure S7.** **circZNF79(5) recruits BRCC3 to remove the K63-linked ubiquitination of YBX1. A**, Huh7 circ-shRNA or -shNC cells transfected with plasmids expressing Flag-YBX1, HA-K48 or HA-K63-linked ubiquitin. The lysates were subjected to immunoprecipitation and polyubiquitination status of YBX1 immunoprecipitated were analyzed by WB. **B**, Huh7 OE-vector or OE-circZNF79(5) cells transfected with plasmids expressing Flag-YBX1 or HA-K63-linked ubiquitin. The lysates were subjected to immunoprecipitation and polyubiquitination status of YBX1 immunoprecipitated were analyzed by WB. **C**, WB analysis of YBX1 stabilization in HCC cells transfected with BRCC36-si2 or si-NC, followed by DMSO, MG132 (20 μM), 3-MA (50 μM) or CHQ (20 μM) treatment for 6 h. **D**, Immunofluorescence displayed that BRCC36 was co-located with YBX1 in Huh7 cells. Scale bars = 20 µm. **E&F**, Endogenous Co-IP using antibody-YBX1 in Huh7 cells transfected with circ-shRNA or shNC (**E**) or SAO (**F**) showed that knocking down circZNF79(5) reduced the association between BRCC36 and YBX1. Three independent experiments with three technical repetitions were performed.

***
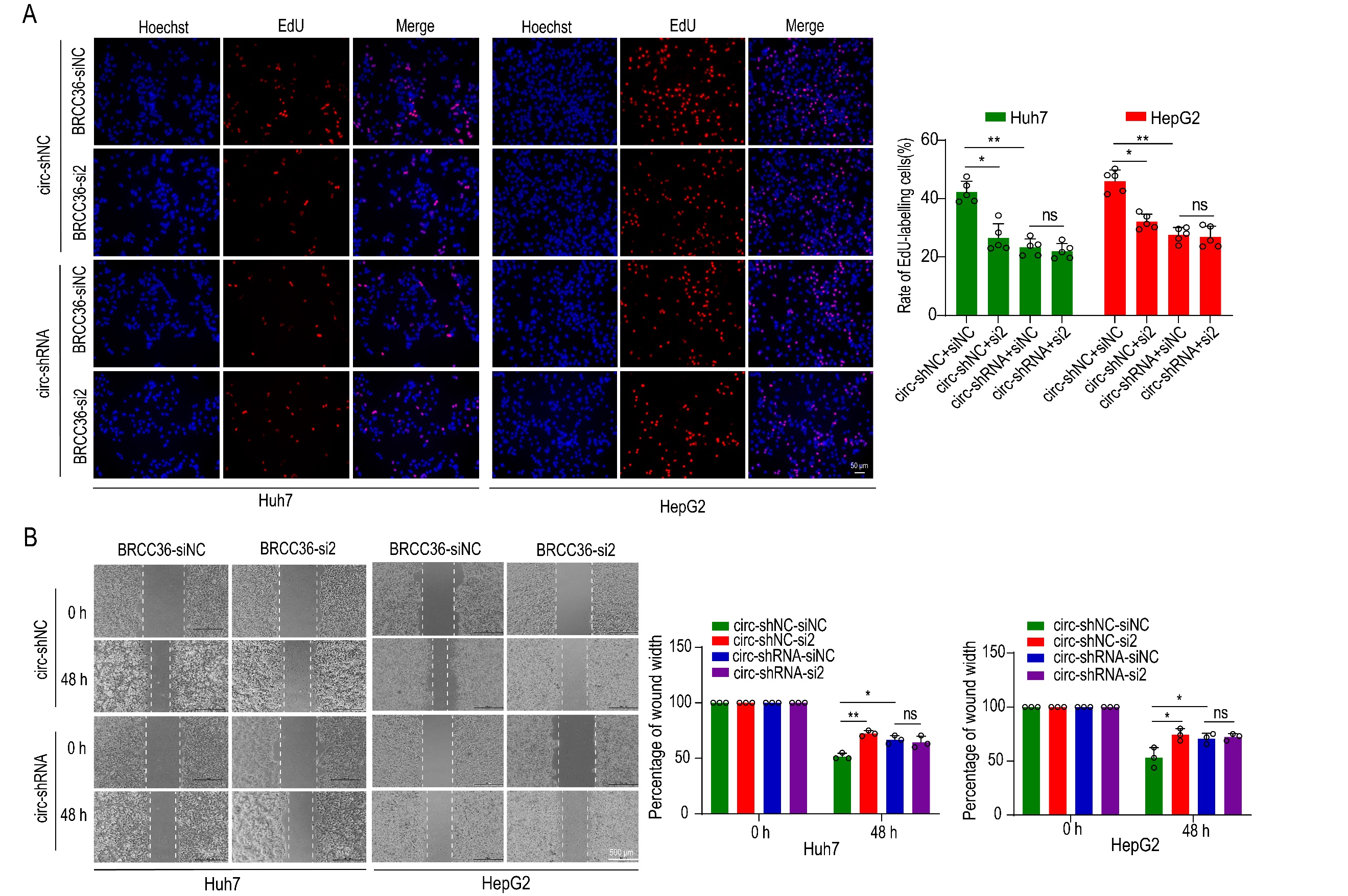
***

**Figure S8. BRCC36's functionality relied on circZNF79(5). A**, EdU assays of Huh7 and HepG2 cells transfected with circ-shRNA or circ-shNC and BRCC36-si2 or siNC, *n* = 5, scale bars = 50 µm. **B**, Cell migration assays of Huh7 and HepG2 cells transfected with circ-shRNA or circ-shNC and BRCC36-si2 or siNC, *n* = 3, scale bars = 500 µm. Statistical analysis was performed using a two-tailed *t*-test. **P* < 0.05, ***P* < 0.01, and *P* > 0.05 not significant (ns). Data are presented as mean ± S.E.M.

**
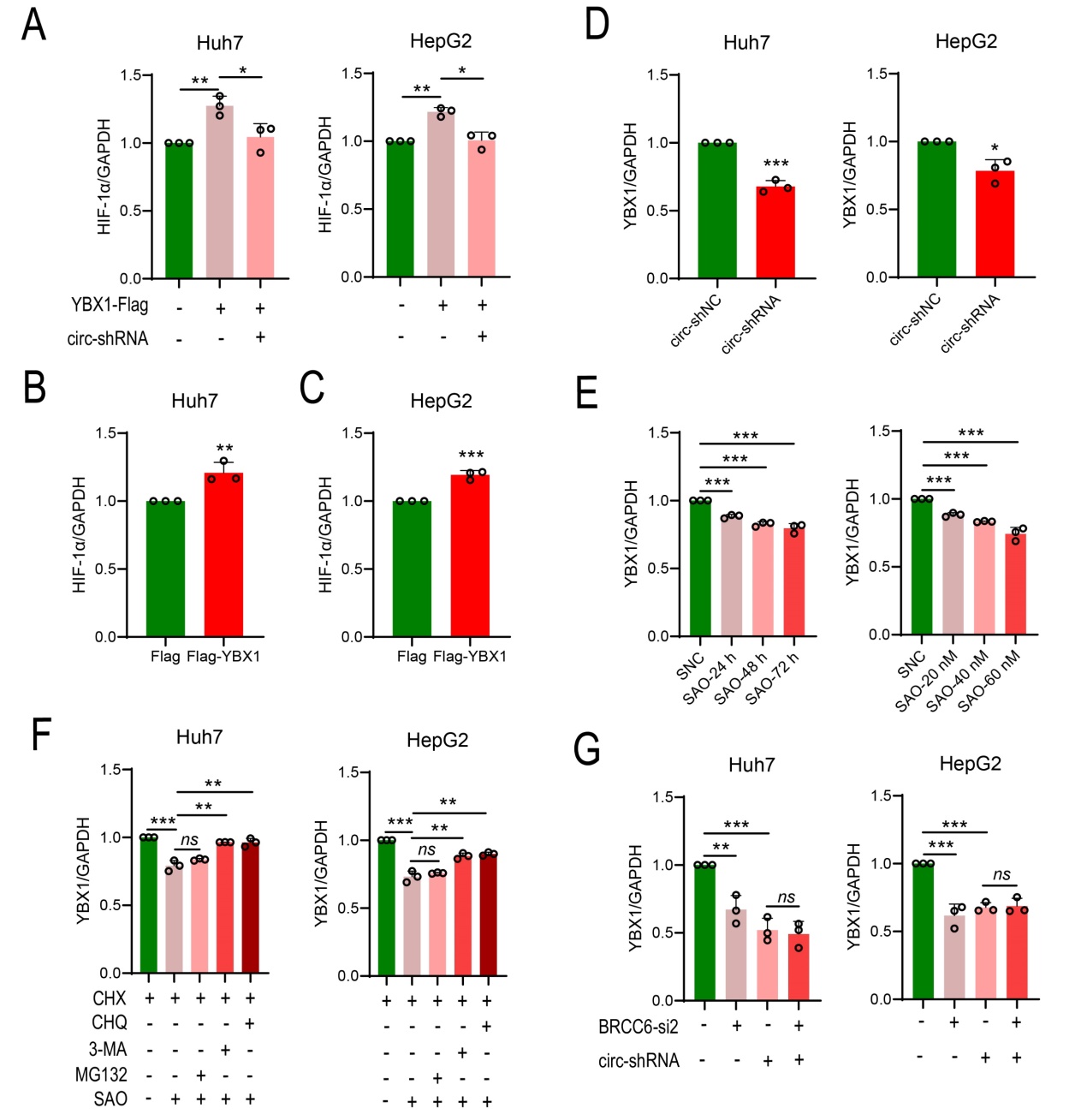
**

**Figure S9. Statistical analysis of partial WB. A**, Fig. 4K. **B**, Fig. 4E. **C**, Fig. S4C. **D**, Fig. 5B. **E**, Fig. S5C. **F**, Fig. S5F**. G**, Fig. 6K.

**
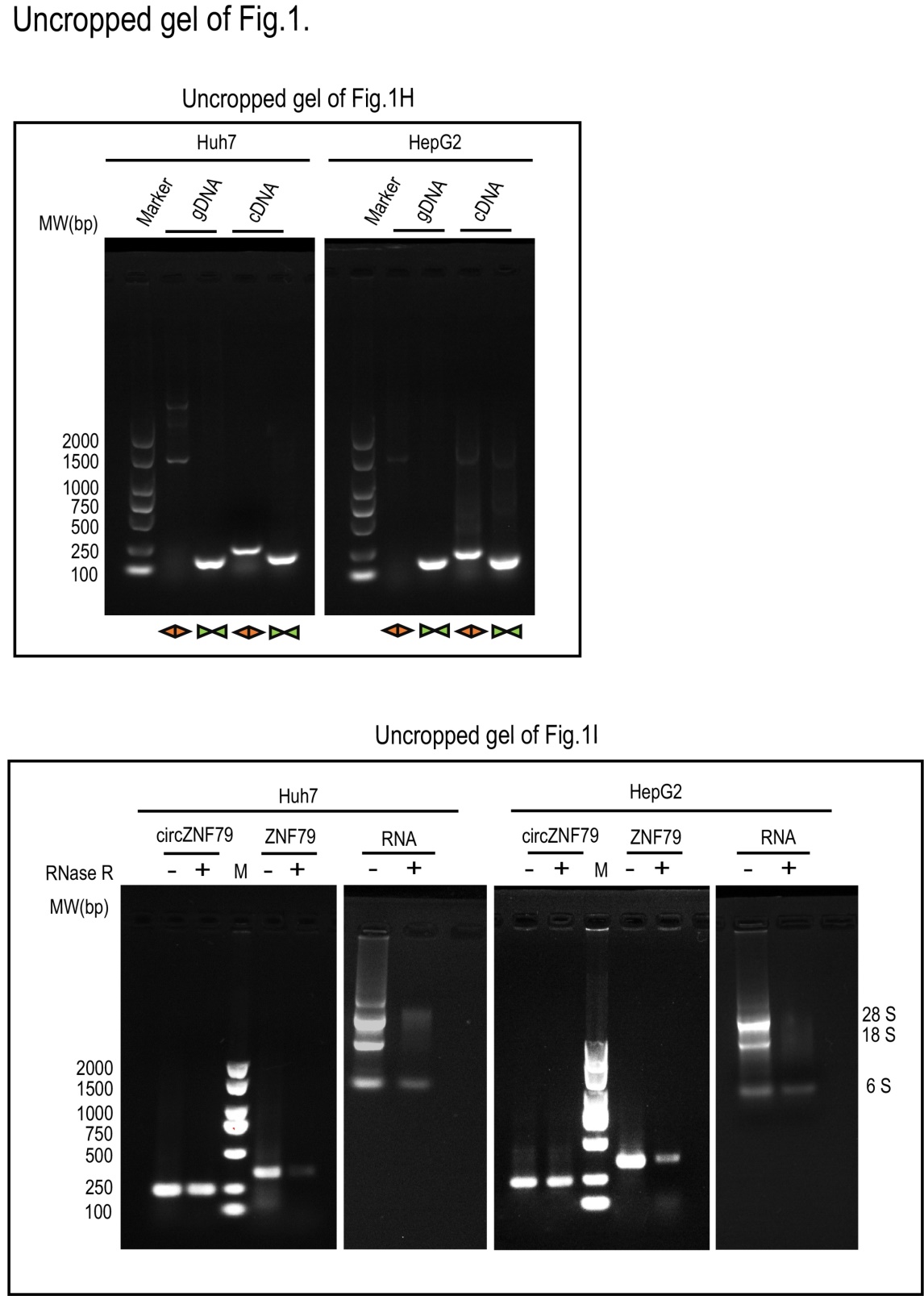
**

**Figure S10. Uncropped blots for Figure 1.**

**
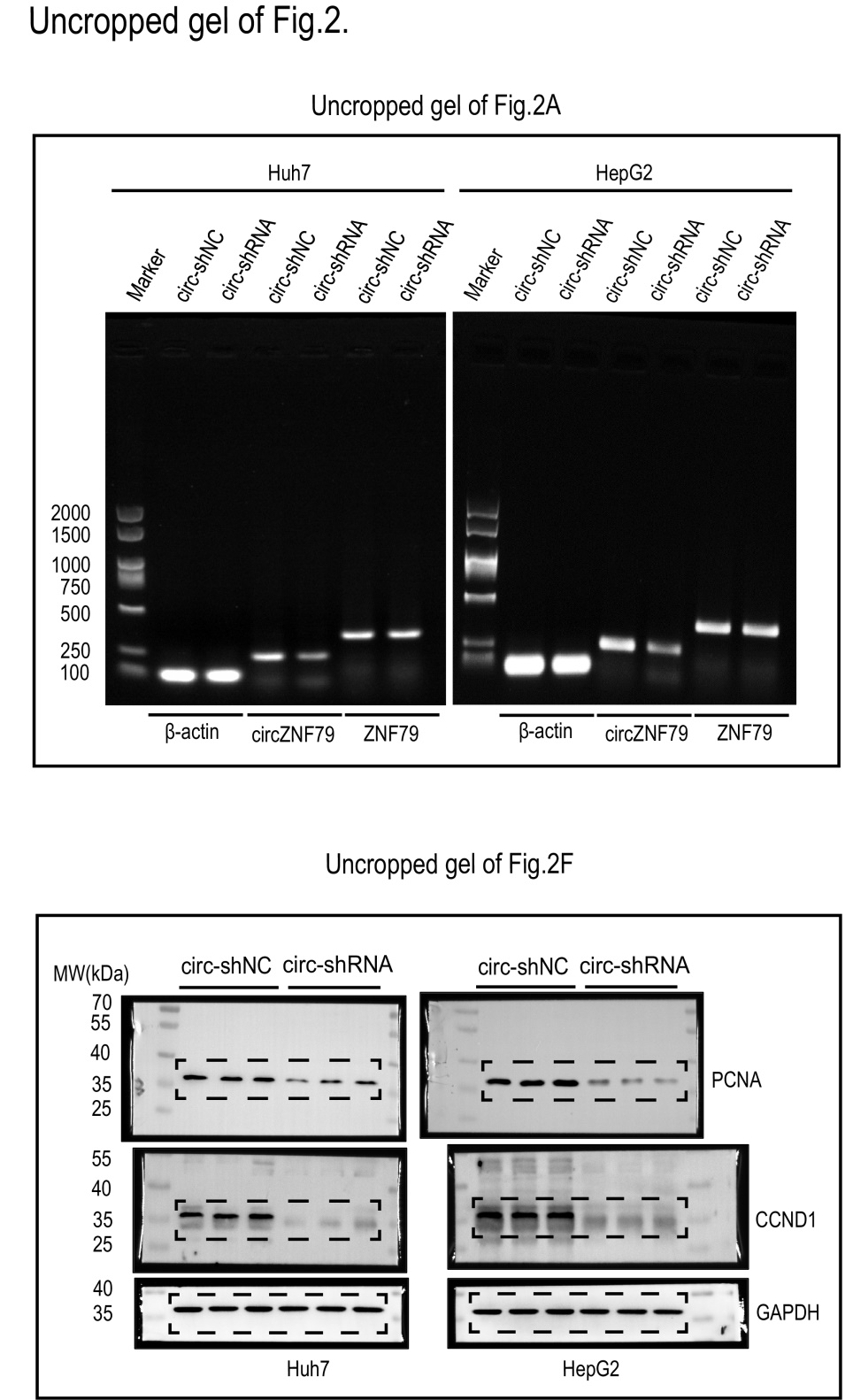
**

**Figure S11. Uncropped blots for Figure 2.**

**
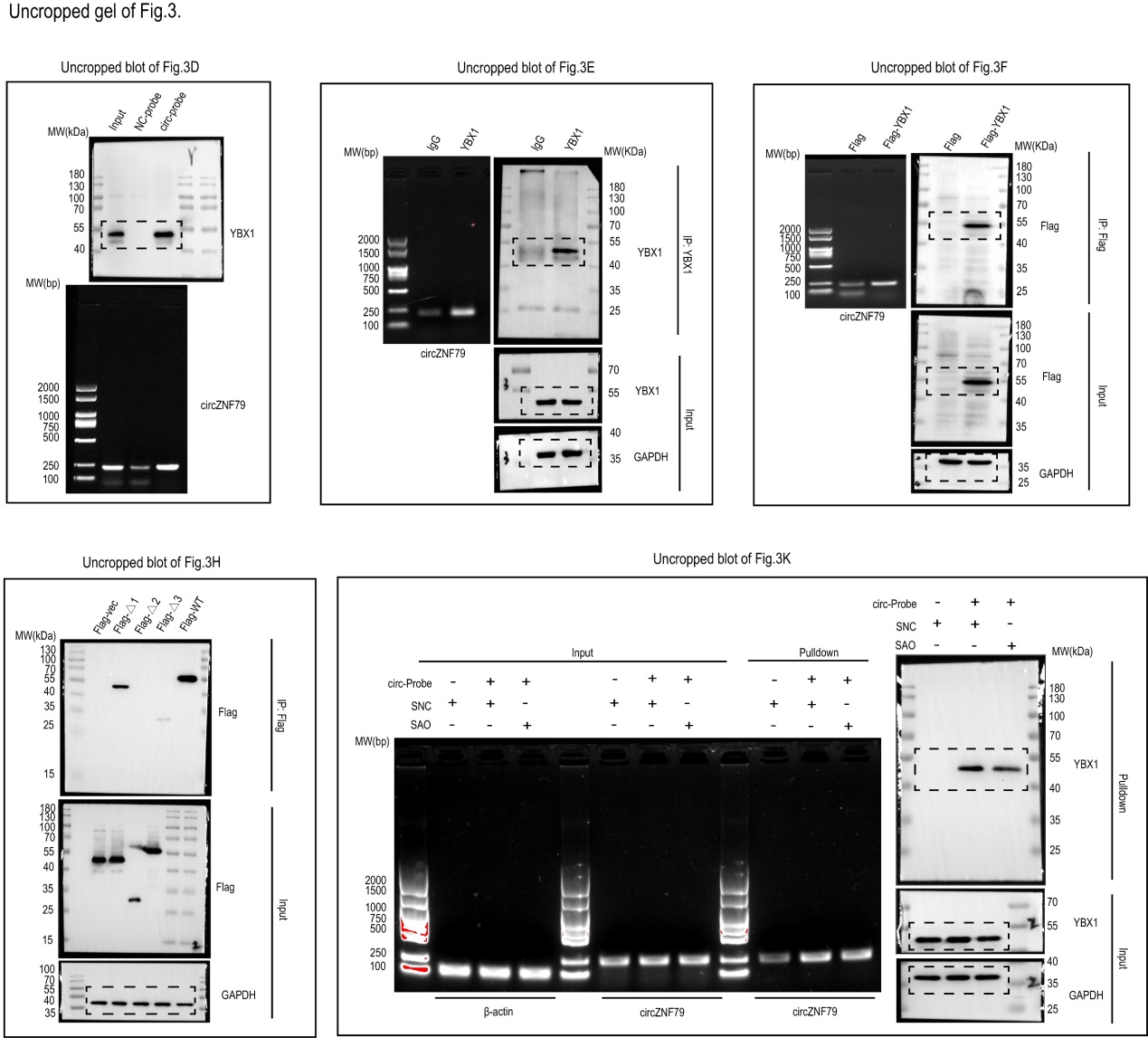
**

**Figure S12. Uncropped blots for Figure 3.**

**
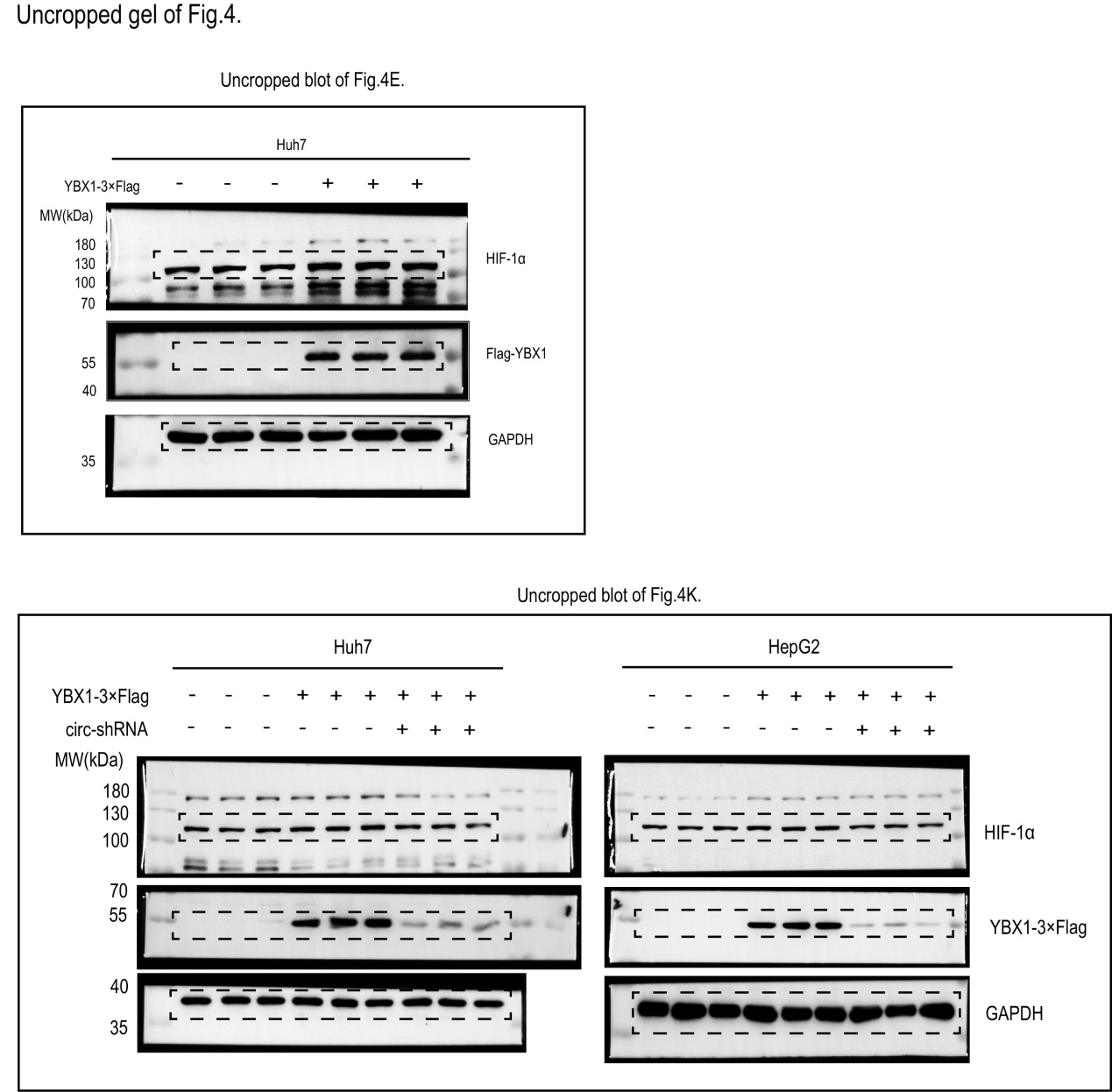
**

**Figure S13. Uncropped blots for Figure 4.**

**
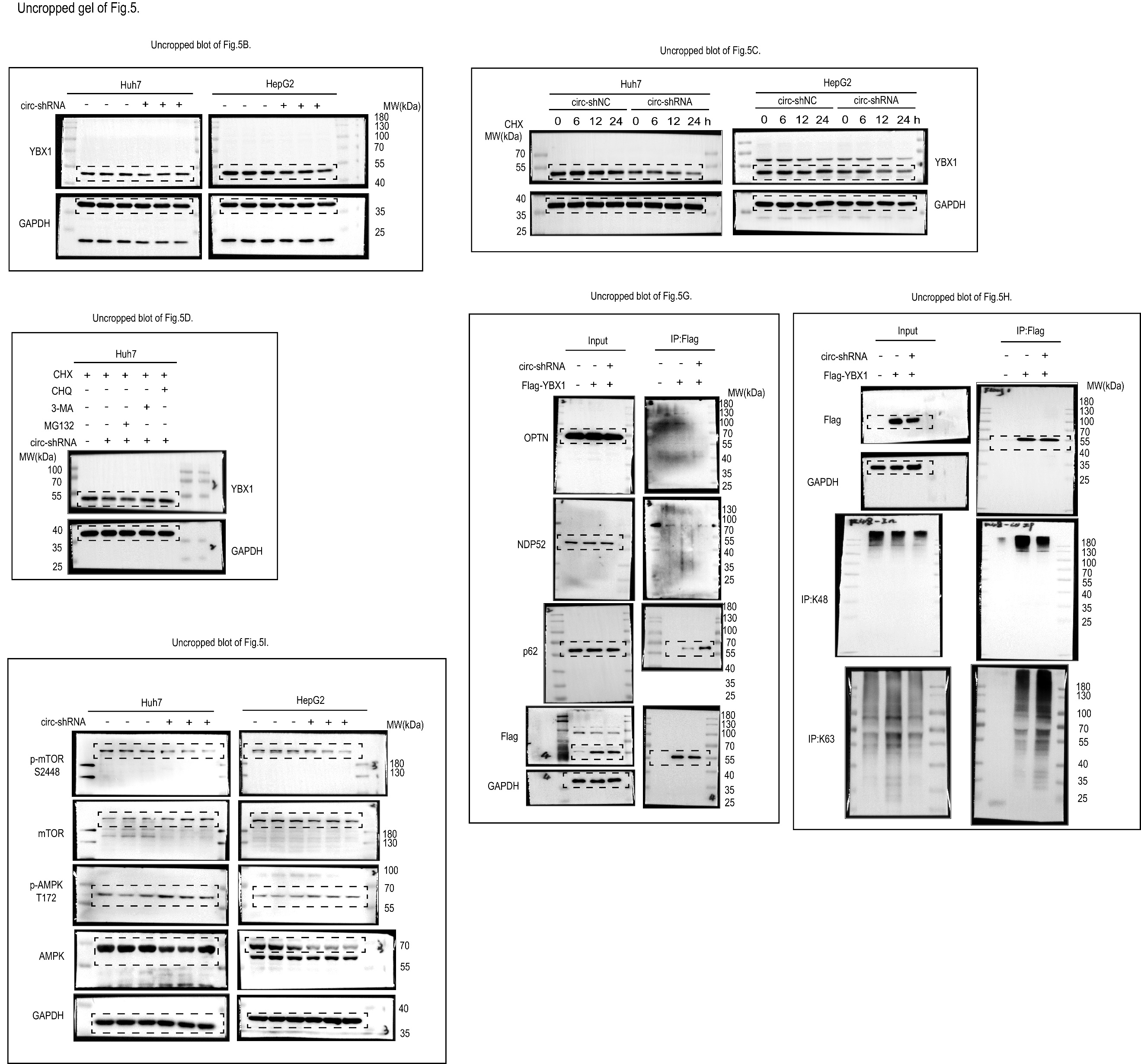
**

**Figure S14. Uncropped blots for Figure 5.**

**
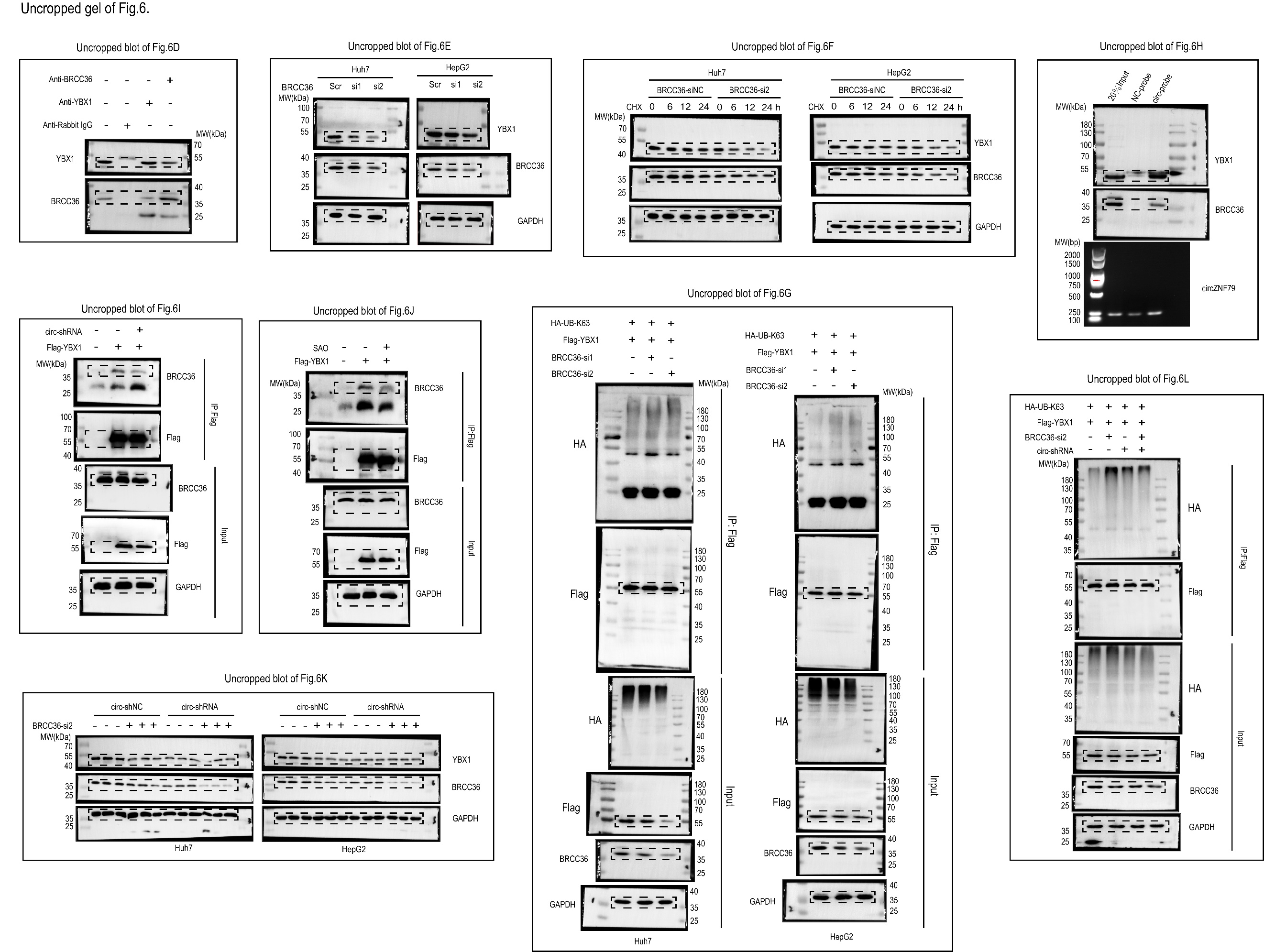
**

**Figure S15. Uncropped blots for Figure 6.**

**
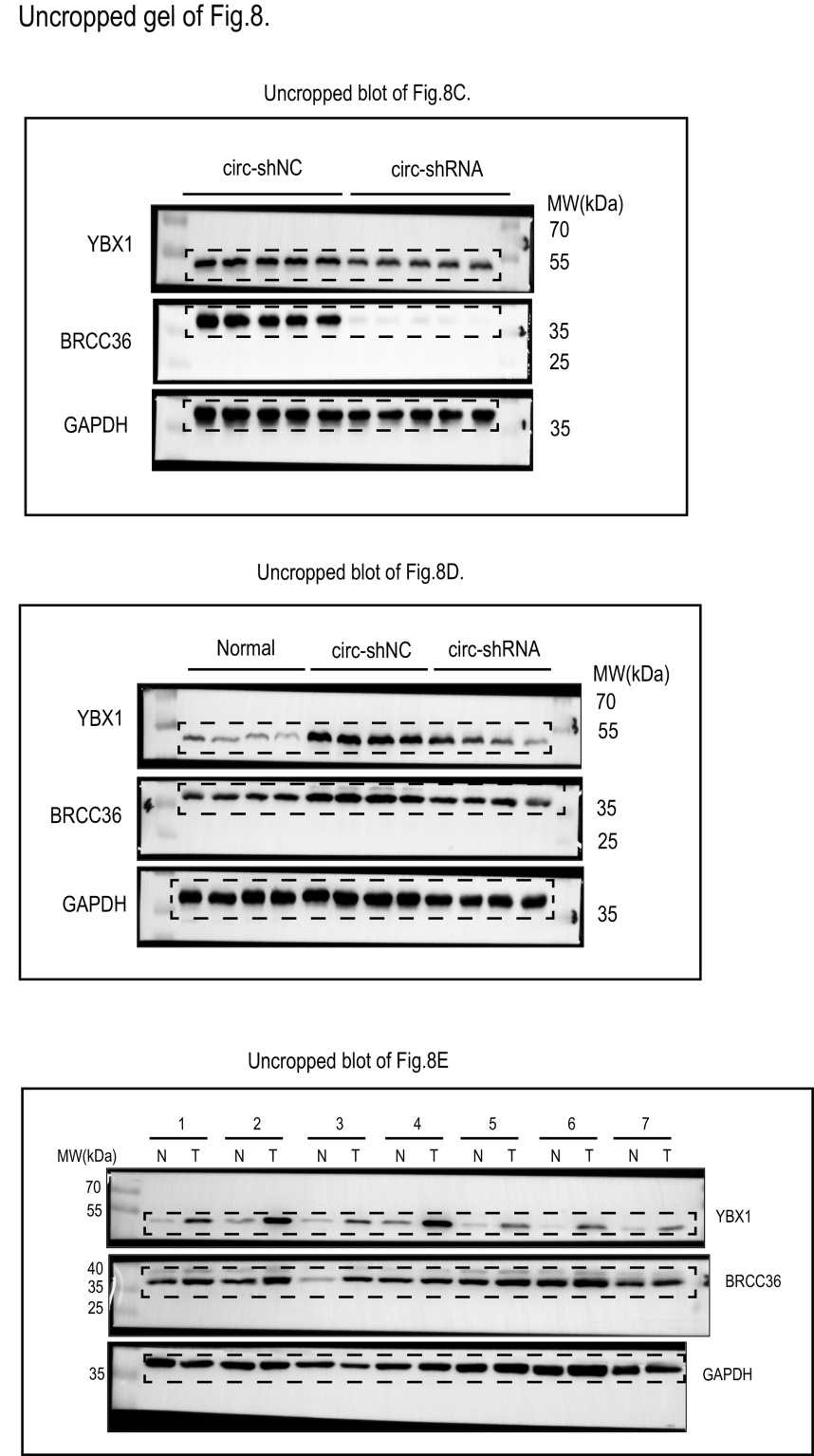
**

**Figure S16. Uncropped blots for Figure 8.**

**
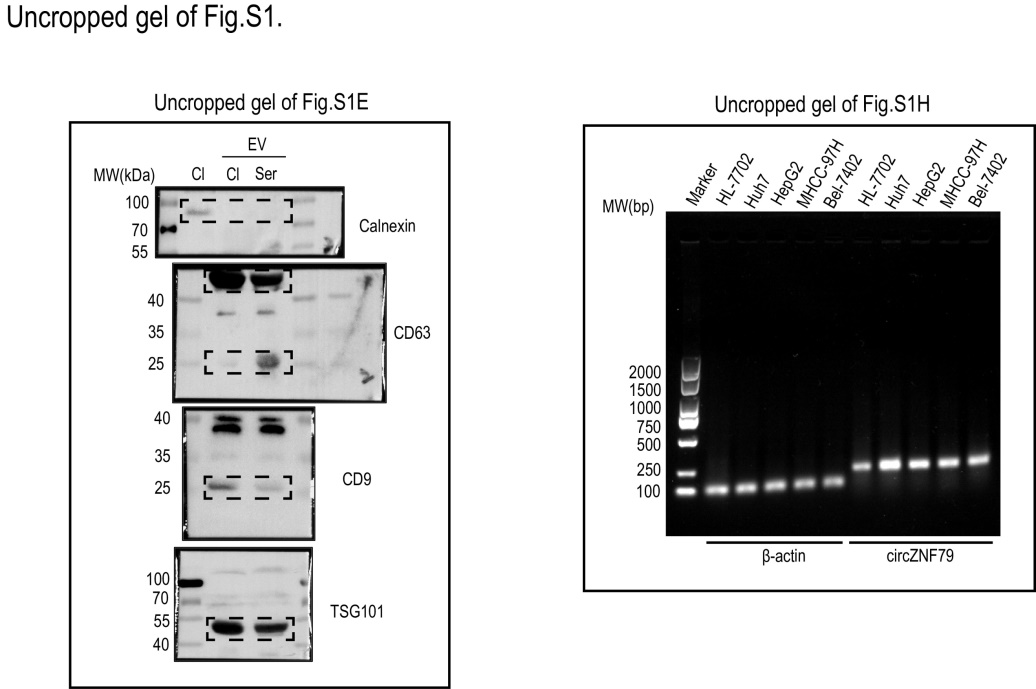
**

**Figure S17. Uncropped blots for Figure S1.**

**
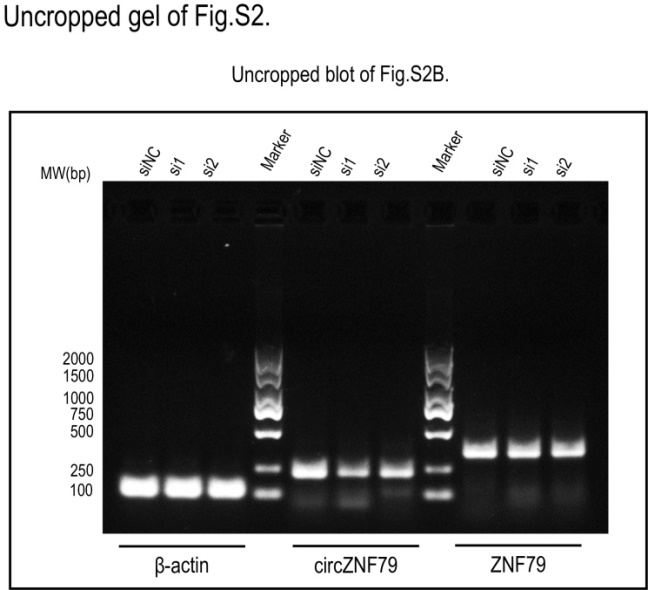
**

**Figure S18. Uncropped blots for Figure S2.**

**
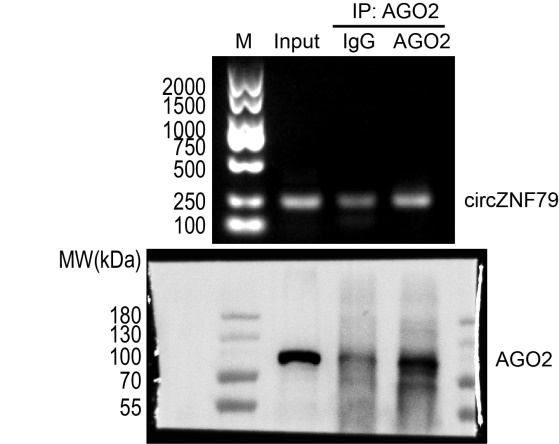
**

**Figure S19. Uncropped blots for Figure S4.**

**
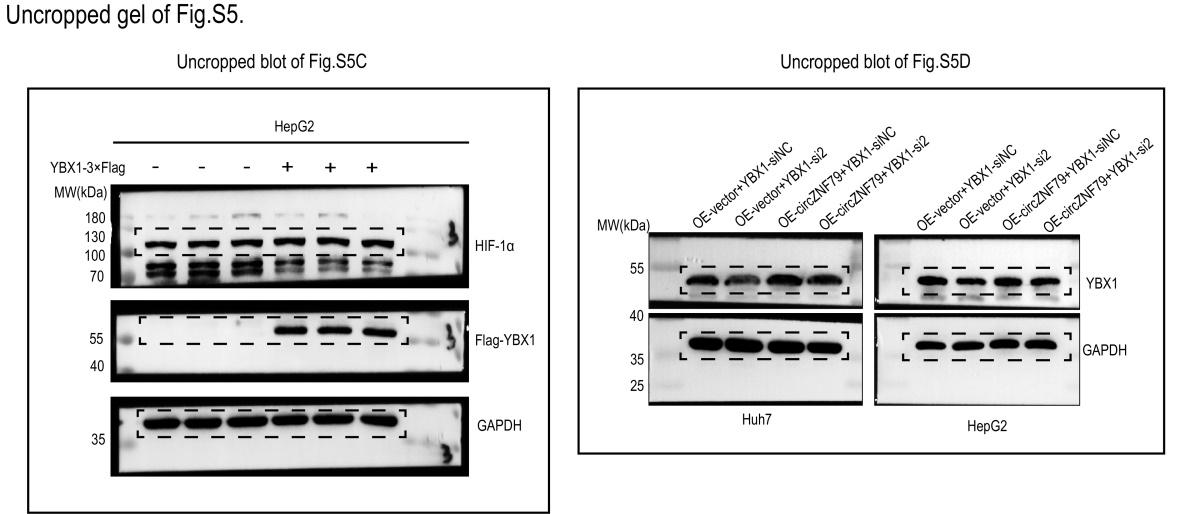
**

**Figure S20. Uncropped blots for Figure S5.**

**
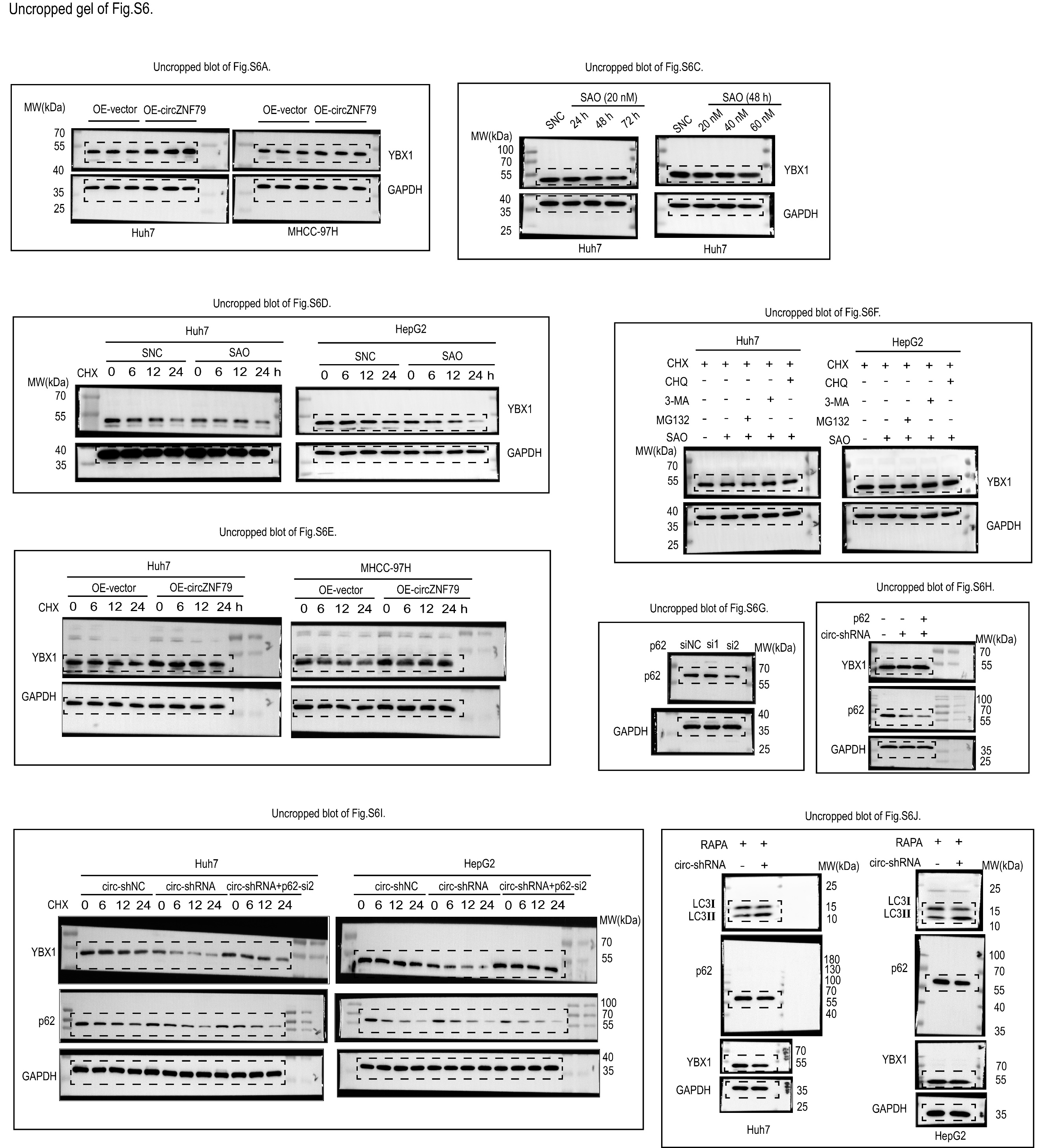
**

**Figure S21. Uncropped blots for Figure S6.**

**
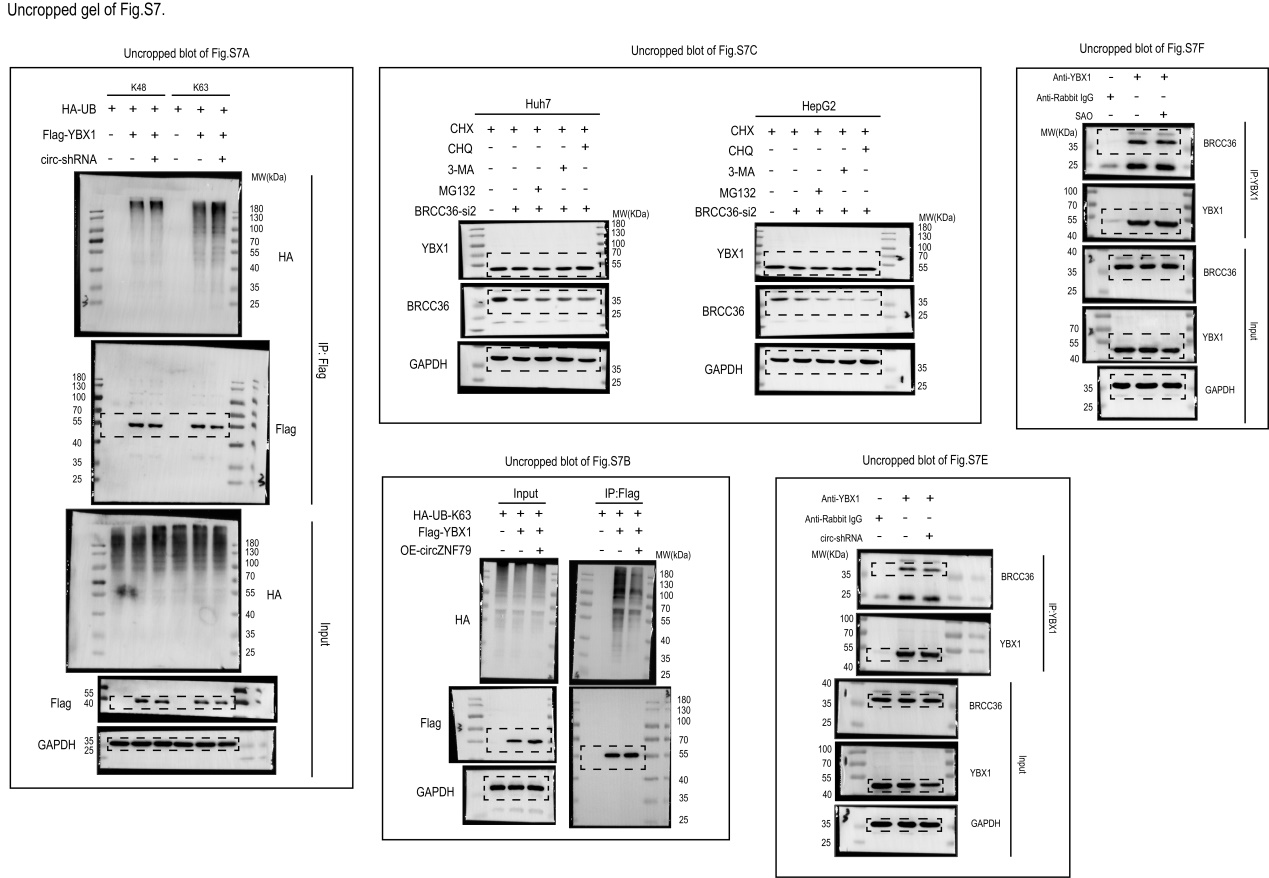
**

**Figure S22. Uncropped blots for Figure S7.**
